# Lepton flavor violation in flavored gauge mediation

**DOI:** 10.1140/epjc/s10052-014-3211-x

**Published:** 2014-12-17

**Authors:** Lorenzo Calibbi, Paride Paradisi, Robert Ziegler

**Affiliations:** 1 Service de Physique Théorique, Université Libre de Bruxelles, 1050 Brussels, Belgium; 2 Dipartimento di Fisica e Astronomia, Universitá di Padova, Via Marzolo 8, 35131 Padua, Italy; 3 INFN Sezione di Padova, Via Marzolo 8, 35131 Padua, Italy; 4 SISSA, Via Bonomea 265, 34136 Trieste, Italy; 5 Sorbonne Universités, UPMC Univ Paris 06, UMR 7589, LPTHE, 75005 Paris, France; 6 CNRS, UMR 7589, LPTHE, 75005 Paris, France

## Abstract

We study the anatomy and phenomenology of lepton flavor violation (LFV) in the context of flavored gauge mediation (FGM). Within FGM, the messenger sector couples directly to the MSSM matter fields with couplings controlled by the same dynamics that explains the hierarchies in the SM Yukawas. Although the pattern of flavor violation depends on the particular underlying flavor model, FGM provides a built-in flavor suppression similar to wave function renormalization or SUSY partial compositeness. Moreover, in contrast to these models, there is an additional suppression of left–right flavor transitions by third-generation Yukawas that in particular provides an extra protection against flavor-blind phases. We exploit the consequences of this setup for lepton flavor phenomenology, assuming that the new couplings are controlled by simple $U(1)$ flavor models that have been proposed to accommodate large neutrino mixing angles. Remarkably, it turns out that in the context of FGM these models can pass the impressive constraints from LFV processes and leptonic electric dipole moments (EDMs) even for light superpartners, therefore offering the possibility of resolving the longstanding muon $g-2$ anomaly.

## Introduction

One of the longstanding problems in particle physics is the origin of flavor hierarchies in the standard model (SM). The most popular attempt to address this problem is in terms of flavor symmetries in which the flavor hierarchies arise from a suitable symmetry breaking pattern. Among the numerous possibilities, the simplest models are based on a single $U(1)$ flavor symmetry [1–4]. In the quark sector this ansatz works pretty well and can account for all hierarchies in quark masses and mixing, with an order-of-magnitude prediction $V_{ub} \sim V_{us} V_{cb}$ that is in good agreement with data. Also in the lepton sector a single $U(1)$ works very well, since charged-lepton mass hierarchies can arise from large charge differences of right-handed leptons, while large mixing angles are due to small charge differences of left-handed leptons. In this way $U(1)$ models can naturally realize the paradigm of an “anarchical” structure [5–8] in lepton mixing, which has recently received renewed attention [9–11] after the reactor neutrino angle $\theta _{13}$ turned out to be sizable.

Independently of the nature of the underlying flavor symmetry, the crucial question about these kind of models regards their predictivity. Since flavor models aim at explaining the origin of dimensionless Yukawa couplings, there is no preferred mass scale of the new degrees of freedom. As new effects in the SM flavor sector are suppressed by this mass scale, there are no observable deviations from the SM flavor predictions, unless this scale is unexpectedly light [12]. Therefore the only way to test these models in laboratory experiments for a high-scale flavor sector is the presence of new physics around the TeV scale, as suggested by the hierarchy problem. If such physics comes with a flavor structure, it can possibly carry down the information of the high-scale flavor sector to the TeV scale and lead to testable predictions for precision flavor observables. The prime example is supersymmetry (SUSY), which in the case of high-scale mediation of SUSY breaking around or above the flavor sector scale directly contains the imprint of the flavor symmetry in the soft-breaking sfermion masses.

However, within the context of gravity mediation simple $U(1)$ models are in big trouble as the suppression of flavor violation is too weak. The reason is that off-diagonal entries in the left-handed and right-handed sfermion mass matrices are suppressed by the differences of the corresponding charges due to their non-holomorphic nature. In the left-handed sector these charge differences are directly related to mixing angles, which for the first two generations are sizable both in the lepton and quark sector. Since the strongest constraints precisely arise from observables involving light families, like $\epsilon _K$ and $\mu \rightarrow e \gamma$, such $U(1)$ flavor models in the context of Gravity Mediation are essentially incompatible with SUSY around the TeV scale.^1^

The situation is completely different in gauge mediation (see Ref. [14]), where the SUSY breaking and the flavor sector can be decoupled. Indeed, if the flavor scale is much higher than the SUSY messenger scale then soft masses are screened from the high-energy flavor sector and have a flavor structure determined only by SM Yukawas, thus realizing the paradigm of minimal flavor violation (MFV) [15]. While this scenario provides a very appealing mechanism to solve the SUSY flavor problem, the imprint of the flavor sector in low-energy physics and thus the possibility to test flavor symmetry models is completely lost.

It is therefore interesting to construct extensions of minimal gauge mediation (MGM) that re-introduce the dependence on the underlying flavor sector, and thus lead to a broad variety of sfermion flavor structures beyond MFV. An example for such extensions is provided by a class of models that has been dubbed “flavored gauge mediation” (FGM) [16]. In these scenarios, new direct couplings between the messengers and the MSSM matter fields are introduced with a flavor structure that is assumed to be controlled by the same underlying flavor symmetry that explains the smallness of the Yukawas.^2^ These couplings generate new contributions to sfermion masses (on top of the flavor-universal MGM ones) that are controlled by the underlying flavor symmetry. Interestingly, due to the loop origin of the soft terms, there is a built-in suppression of flavor violation that is independent of the underlying flavor model [28]. This implies that even single $U(1)$ flavor models are perfectly viable (in contrast to gravity mediation), as the flavor pattern of the resulting sfermion masses resembles the suppression in wave function renormalization [29, 30] or SUSY partial compositeness [31–34]. Moreover, in contrast to those scenarios, there is also a built-in suppression of LR flavor transitions and in particular flavor-blind phases by third-generation Yukawas, which becomes very efficient in the down and charged-lepton sector provided $\tan \beta$ is not very large.

While in Ref. [28] we have focused on the quark sector, in this paper we analyze the impact of FGM models with underlying $U(1)$ flavor models on the lepton sector. There are good arguments that motivate this study: (i) in contrast to the quark sector the large neutrino mixing angles require milder hierarchies in left-handed charges, leading in turn to weaker suppression in the left-handed slepton sector and therefore potentially large effects in LFV processes, (ii) the experimental bounds on LFV channels with an underlying $\mu \rightarrow e$ transition as well as the electron EDM underwent recently a very significant improvement challenging many models with new physics (NP) at the TeV scale, even with modest sources of flavor violation. Therefore the major aim of this work is to analyze whether and to which extent single $U(1)$ flavor models for the lepton sector are viable in the context of FGM. A related question is whether we can account for the current muon $g-2$ anomaly, that is, if light sleptons are still allowed by the LFV and EDM bounds (for a general discussion on the interrelationship of leptonic dipoles see Ref. [35]).

The rest of the paper is organized as follows: in Sect. 2 we recall the main ingredients of FGM models providing explicit expressions for the soft masses in the slepton sector. Concrete examples of $U(1)$ leptonic flavor models and their imprint in the soft sector are presented in Sect. 3. The low-energy phenomenology of FGM models supplemented by the above $U(1)$ flavor models is studied in Sect. 4. In Sect. 5, we compare the flavor structure of the soft terms and related phenomenological implications of FGM models to $U(1)$ models with gravity mediation and models with SUSY partial compositeness. We conclude in Sect. 6. In an appendix we collect the formulas for LFV branching ratios, lepton anomalous magnetic moments and lepton electric dipole moments using a generalized mass insertion approximation without assuming large $\tan \beta$, thus improving on existing results that take into account only the $\tan \beta$ enhanced terms.

## Flavored gauge mediation

We begin with a brief review of MGM (see Ref. [14]). In this scenario $N$ copies of heavy chiral superfields $\Phi _i + \overline{\Phi }_i$ in $\mathbf{5+\overline{5}}$ of SU(5) are introduced. These messenger fields couple directly to the SUSY breaking sector, which is effectively parameterized by a single spurion field $X$ that gets a vev $\langle X \rangle = M + F \theta ^2$. Through the following superpotential coupling

1 $$\begin{aligned} W \supset X \overline{\Phi }_i \Phi _i, \quad i=1 \ldots N, \end{aligned}$$

the messengers acquire large supersymmetric mass terms $M$ and SUSY breaking masses proportional to $F$. By integrating out the messengers at loop level, soft terms are generated. At the messenger scale, A-terms vanish and gaugino masses and sfermion masses are given by

2 $$\begin{aligned} M_i(M)&= N \frac{\alpha _i(M)}{4 \pi } ~\Lambda , \qquad \Lambda = \frac{F}{M}, \end{aligned}$$

3 $$\begin{aligned} m^2_{\tilde{f}}(M)&= 2 N \sum _{i=1}^3 C_i(f)~ \frac{\alpha ^2_i(M)}{(4 \pi )^2} ~\Lambda ^2, \qquad f=q,\,u,\,d, \ldots , \end{aligned}$$

where $C_i(f)$, $i=1,2,3$ is the quadratic Casimir of the representation of the field $f$ under the gauge group SU(3) $\times$ SU(2) $\times$ U(1).

Since the messengers have the same gauge quantum numbers as the MSSM Higgs fields, in addition to the Yukawa couplings

4 $$\begin{aligned} W \supset (y_U)_{ij} Q_i U_j H_u + (y_D)_{ij} Q_i D_j H_d + (y_E)_{ij} L_i E_j H_d,\nonumber \\ \end{aligned}$$

also direct couplings of messengers to MSSM fields are allowed by the gauge symmetries. If we restrict to R-parity even messenger fields,^3^ the messengers can couple only to the MSSM matter fields. For the messenger doublets these couplings read in general

5 $$\begin{aligned} \Delta W&= (\lambda _U)_{ij} Q_i U_j \Phi _{H_u} + (\lambda _D)_{ij} Q_i D_j \overline{\Phi }_{H_d}\nonumber \\&\quad + (\lambda _E)_{ij} L_i E_j \overline{\Phi }_{H_d}, \end{aligned}$$

where $\Phi _{H_u}, \overline{\Phi }_{H_d}$ denote the SU(2) doublet components of the $\mathbf{5}, {\overline{\mathbf{5}}}$ messengers, and we restricted to the case of one messenger pair for simplicity.

The presence of direct messenger-matter couplings gives rise to new contributions to sfermion masses and A-terms with a flavor structure that depends on the new parameters $\lambda _{ij}$. If these couplings were flavor-anarchic ${\mathcal {O}}\left( 1 \right)$ numbers, the elegant solution of Gauge Mediation to the SUSY flavor problem would be completely spoiled. Therefore it is usually assumed that all direct couplings of the messengers to matter fields vanish, which can be enforced for example by introducing a new $Z_2$ symmetry under which MSSM fields are even and messengers are odd. Note that this symmetry extends to a full accidental $U(1)$ symmetry in the case of one messenger pair

6 $$\begin{aligned} \Phi \rightarrow e^{i \alpha } \Phi , \quad \overline{\Phi } \rightarrow e^{- i \alpha } \overline{\Phi }. \end{aligned}$$

However, in order to preserve the neat solution of the SUSY flavor problem in MGM, it is enough that the new couplings in Eq. (5) are just sufficiently small. Such small couplings can easily be motivated in the context of flavor models, since they break the global flavor symmetries of MSSM kinetic terms exactly as the Yukawas, and therefore they can naturally have a similar hierarchical structure. This can be realized in explicit flavor models in which the messenger fields transform like the Higgs fields (in particular one can choose that they do not transform at all under the flavor sector), which implies that the new couplings have the same parametric suppression as the Yukawas,

7 $$\begin{aligned} \lambda _U \sim y_U, \quad \lambda _D \sim y_D, \quad \lambda _E \sim y_E. \end{aligned}$$

Following Ref. [16], we refer to these kind of models as FGM.

The new contributions to soft terms induced by the couplings in Eq. (5) can be calculated using the general expressions in Ref. [25]. At leading order in SUSY breaking one finds new contributions to sfermion masses at 2-loop and non-vanishing A-terms at 1-loop. While these new effects can have interesting consequences for the low-energy spectrum [36, 37], here we are mainly interested in the flavor structure of the new contributions to sfermion masses, in particular in the slepton sector. Therefore we will now take a bottom-up point of view and restrict the analysis to the consequences of the presence of the $\lambda _E$ coupling for the lepton sector. We will not discuss the impact of other possible messenger-matter couplings on the low-energy spectrum, in particular the mass of the lightest Higgs boson. We just note that the Higgs mass does not represent a serious constraint in these kind of models, and can be due to large A-terms or an implementation in the NMSSM. The latter also represents a natural possibility to generate the $\mu$-term and to elegantly solve the $\mu$–$B_\mu$ problem of Gauge Mediation, since in the NMSSM the general structure of FGM motivates a direct coupling of the NMSSM singlet to the messengers which can easily allow for correct EWSB [21, 38].

Furthermore, let us notice that the couplings $\lambda _E$ do not deform the spectrum predicted by the underlying gauge mediation scheme, at least for low to moderate values of $\tan \beta$, as we are going to consider in the next sections. In particular, if $m_h\approx 126$ GeV is accounted for by a large top A-term, induced by an $\mathcal {O}(1)$ coupling $(\lambda _U)_{33}$ in Eq. (5), the spectrum would resemble the one discussed e.g. in Ref. [28]. This would have interesting consequences for the leptonic sector we consider here, since $(\lambda _U)_{33} = \mathcal {O}(1)$ also suppresses the masses of the left-handed sleptons, through an induced Fayet–Iliopoulos term, thus naturally accomodating the Higgs mass with a light slepton spectrum that can give a sizable contribution to the muon $g-2$ [19, 28].

For soft terms in the slepton sector we use the conventions

8 $$\begin{aligned} \mathcal{L}&\supset - \left( (\tilde{m}^2_L)_{ij} L_i L_j^\dagger + (\tilde{m}^2_E)_{ij} E_i^\dagger E_j + (A_e)_{ij} L_i E_j H_d \right) |_{scalar} \nonumber \\&= - \left( \tilde{l}_L^T \tilde{m}^2_L \tilde{l}_L^* + \tilde{e}_R^T (\tilde{m}^2_E)_{ij} \tilde{e}_R^* + \tilde{l}_L^T A_e \tilde{e}_R^* H_d \right) , \end{aligned}$$

where the first line denotes the scalar components of superfields. Using the results of Ref. [25], the presence of $\lambda _E$ gives rise to the following expressions for the non-holomorphic masses^4^:

9 $$\begin{aligned}&{\tilde{m}}^2_{L} = \frac{\Lambda ^2}{256 \pi ^4} \bigg [ N \left( \frac{3}{2} g_2^4 + \frac{3}{10} g_1^4 \right) -\left( \frac{9}{5}g_1^2 + 3 g_2^2 \right) \lambda _E\lambda _E^\dagger \nonumber \\&\quad +\, 3 \lambda _E\lambda _E^\dagger \lambda _E\lambda _E^\dagger + 2\lambda _Ey_E^\dagger y_E\lambda _E^\dagger -2y_E\lambda _E^\dagger \lambda _Ey_E^\dagger \nonumber \\&\quad +\, \lambda _E\lambda _E^\dagger \mathrm{Tr} \left( \lambda _E\lambda _E^\dagger \right) + y_E\lambda _E^\dagger \mathrm{Tr} \left( \lambda _Ey_E^\dagger \right) + \lambda _Ey_E^\dagger \mathrm{Tr} \left( y_E\lambda _E^\dagger \right) \bigg ],\nonumber \\ \end{aligned}$$

and

10 $$\begin{aligned} {\tilde{m}}^2_E&= \frac{\Lambda ^2}{256 \pi ^4} \bigg [ \frac{6}{5}g_1^4 N -\left( \frac{18}{5}g_1^2 + 6 g_2^2 \right) \lambda _E^\dagger \lambda _E+ 6 \lambda _E^\dagger \lambda _E\lambda _E^\dagger \lambda _E\nonumber \\&+ 2 \lambda _E^\dagger y_Ey_E^\dagger \lambda _E- 2 y_E^\dagger \lambda _E\lambda _E^\dagger y_E+ 2\lambda _E^\dagger \lambda _E\mathrm{Tr} \left( \lambda _E\lambda _E^\dagger \right) \nonumber \\&+ 2 \lambda _E^\dagger y_E\mathrm{Tr} \left( \lambda _Ey_E^\dagger \right) + 2 y_E^\dagger \lambda _E\mathrm{Tr} \left( y_E\lambda _E^\dagger \right) \bigg ], \end{aligned}$$

while the A-terms are given by

11 $$\begin{aligned} A_E = -\frac{\Lambda }{16 \pi ^2} \left( \lambda _E\lambda _E^\dagger y_E+ 2y_E\lambda _E^\dagger \lambda _E\right) . \end{aligned}$$

Note that the flavor dependence of the above expressions can be obtained using a simple spurion analysis, taking into account also the $U(1)_M$ “messenger number” in Eq. (6) as a spurious symmetry under which the new couplings are charged. The $U(1)_M$ symmetry prevents terms like $\lambda _Ey_E^\dagger$ and $\lambda _E^\dagger y_E$ for the non-holomorphic masses ${\tilde{m}}^2_{L}$ and ${\tilde{m}}^2_{E}$, respectively, and terms like $\lambda _E$, $\lambda _Ey_E^\dagger \lambda _E$ and $\lambda _E^\dagger y_E\lambda _E^\dagger$ for the A-terms. As a result, the A-terms are partially aligned to the Yukawa couplings and their diagonal components are necessarily real and therefore do not induce contributions to the EDMs.

For future convenience, we define the flavor violating mass insertions (MIs) as usual

12 $$\begin{aligned} (\delta _{LL}^e)_{ij}&= \frac{({\tilde{m}}^2_{L})_{ij}}{{\tilde{m}}^2_{L}}~, \quad (\delta _{RR}^e)_{ij} = \frac{({\tilde{m}}^2_{E})_{ij}}{{\tilde{m}}^2_{E}}~,&\nonumber \\&(\delta _{LR}^e)_{ij} = \frac{v_d(A_{E})_{ij}}{{\tilde{m}}_{L}{\tilde{m}}_{E}}~. \end{aligned}$$

In the limit of $y_E,\lambda _E\ll 1$, i.e. for moderate/low $\tan \beta$ values, we obtain the following approximate expressions at the messenger scale:

13 $$\begin{aligned}&(\delta _{LL}^e)_{ij} \simeq - \left( \frac{10 g_2^2 + 6 g_1^2}{5 g_2^4 + g_1^4}\right) \frac{(\lambda _E\lambda _E^\dagger )_{ij}}{N}, \nonumber \\&(\delta _{RR}^e)_{ij}- \left( \frac{5 g_2^2 + 3 g_1^2}{g_1^4}\right) \frac{(\lambda _E^\dagger \lambda _E)_{ij}}{N},\end{aligned}$$

14 $$\begin{aligned}&(\delta _{LR}^e)_{ij} \simeq - \frac{1}{\sqrt{\frac{3N}{\sqrt{5}}} g_1g_2}~ \frac{m^e_j (\lambda _E\lambda _E^\dagger )_{ij} + 2 m^e_i (\lambda _E^\dagger \lambda _E)_{ij}}{\sqrt{\tilde{m}_L \tilde{m}_E}}. \end{aligned}$$

Few comments are in order:

- The above MIs, as well as all superpotential couplings, are defined in the basis where we define the flavor model. In order to study their phenomenological consequences, we go to the mass basis for the charged-lepton Yukawas by means of the rotation $y_E \rightarrow V_{EL}^T y_E V_{ER} = y_E^\mathrm{diag}$. Under this change of basis, the spurion $\lambda _E$ transforms accordingly. However, one can easily check that in $U(1)$ models with non-negative charges the parametric flavor suppression remains the same and only the $\mathcal{O}(1)$ coefficients change. We therefore simply ignore these differences, that is we take $V_{EL}^T \lambda _E V_{ER} \sim \lambda _E$.
- Interestingly, the diagonal A-terms are real. As a result, the leading CP violating phases generating the EDMs can only arise at higher order in the MIs, through the combination $(\delta _{LL}^e)_{ik} (\delta _{LR}^e)_{kj}$, $(\delta _{LR}^e)_{ik} (\delta _{RR}^e)_{kj}$, and $(\delta _{LL}^e)_{ik} (\delta _{LR}^e)_{kk} (\delta _{RR}^e)_{kj}$ when $ij=11$. This, however, leads to an additional suppression by powers of $(\lambda _E)_{33} \sim y_\tau$.^5^
- As a consequence of Eqs. (13), (14), the naive expectations for the MIs are enhanced, for a given number of messengers $N$, by large (mediation-scale dependent) gauge factors. This is especially true in the case of $(\delta _{RR}^e)_{ij}$ and, to less extent, also in the cases of $(\delta _{LL}^e)_{ij}$ and $(\delta _{LR}^e)_{ij}$.

In the following, we will analyze the impact of our FGM model on the branching ratio of $\mu \rightarrow e \gamma$ and the electron EDM which are the most powerful probes of new physics in the leptonic sector. To do so, we need to specify the underlying flavor model that controls the flavor structure of the new couplings.

## Flavored gauge mediation and $U(1)$ flavor models

While the results of the last section can be applied to any flavor model that predicts the flavor structure of $y_E$ and therefore $\lambda _E$, in this section we concentrate on simple $U(1)$ flavor models. We first recall the basic structure of these models, then we analyze their predictions for the soft terms in the lepton sector in the context of our FGM model.

### $U(1)$ Flavor models

In the simplest realization of these models the flavor symmetry is spontaneously broken by the vev of a single “flavon” field with negative unit charge. Yukawa couplings then arise from higher-dimensional operators that involve suitable powers of the flavon to make the operator invariant under the $U(1)$ symmetry, with some undetermined coefficients that are assumed to be $\mathcal{O}(1)$. The suppression scale is the typical scale of the flavor sector that could correspond to the mass scale of Froggatt–Nielsen messengers in explicit UV completions. The Yukawas then depend only on powers of the ratio $\epsilon$ of flavon vev and flavor scale, which typically is taken to be of the order of the Cabibbo angle $\epsilon \sim 0.2$. If we restrict to models where only the matter fields are charged, i.e. $H_u = H_d = 0$, we get for the lepton Yukawa couplings

15 $$\begin{aligned} (y_E)_{ij} \sim \epsilon ^{L_i + E_j}, \end{aligned}$$

where $L_i$ and $E_i$ stand for the $U(1)$ charges of the left-handed and right-handed leptons, respectively. The neutrino sector depends on the origin of neutrino masses. If neutrinos are Dirac, then the Yukawa coupling takes the same form as the charged-lepton Yukawa above with $E_j \rightarrow N_j$. In this case, the left-handed rotations $V_{EL}, V_{NL}$ for the charged-lepton and neutrino sectors, respectively, and therefore the PMNS matrix $V_{PMNS}$, have the same parametric structure

16 $$\begin{aligned} (V_{PMNS})_{ij} \sim (V_{EL})_{ij} \sim (V_{NL})_{ij} \sim \epsilon ^{|L_i - L_j|}. \end{aligned}$$

Large neutrino mixing angles can therefore be reproduced by taking small left-handed charge differences $L_i-L_j$. Instead small neutrino masses can be accommodated by taking sufficiently large charges $N_i$ of right-handed neutrinos. A more plausible explanation of light neutrinos can be achieved if they originate from the Weinberg operator

17 $$\begin{aligned} \Delta W = \frac{(y_{ll})_{ij}}{\Lambda } L_i L_j H_u H_u, \end{aligned}$$

with a flavor structure determined by the $U(1)$ symmetry

18 $$\begin{aligned} (y_{ll})_{ij} \sim \epsilon ^{L_i + L_j}. \end{aligned}$$

In this way the smallness of neutrino masses can be elegantly explained by assuming a large UV scale $v_u/\Lambda \ll 1$, but the prediction for the parametric structure of the left-handed neutrino rotations and therefore for the PMNS matrix does not change, and we still get the result of Eq. (16). One possibility for an explicit UV completion is the type-I seesaw mechanism. In this scenario, one adds three heavy right-handed neutrinos and Dirac Yukawa couplings

19 $$\begin{aligned} \Delta W = (y_\nu )_{ij} L_i N_j H_u + \frac{1}{2} (M_N)_{ij} N_i N_j, \end{aligned}$$

with their flavor structure given by

20 $$\begin{aligned} (y_\nu )_{ij} \sim \epsilon ^{L_i + N_j} \quad (M_N)_{ij} \sim M_N \epsilon ^{N_i + N_j}. \end{aligned}$$

Integrating out the right-handed neutrinos generates the Weinberg operator with a coefficient given by

21 $$\begin{aligned} \frac{(y_{ll})_{ij}}{\Lambda } = - \frac{1}{2} (y_\nu M_N^{-1} y_\nu ^T)_{ij}. \end{aligned}$$

Note that in the simple $U(1)$ models that we will consider here, the parametric flavor structure of the coefficient of the Weinberg operator is the same as in the effective theory

22 $$\begin{aligned} (y_{ll})_{ij} \sim \epsilon ^{L_i + L_j}, \end{aligned}$$

and therefore we recover the same estimate for the PMNS matrix as in Eq. (16).

Various $U(1)$ models have been discussed in the literature; see e.g. [6, 10, 39, 40]. There is some ambiguity in the choice of charge assignments, since $\epsilon$ is typically not a very small parameter (one has $\epsilon \approx 0.2 \div 0.5$) so that the unknown $\mathcal{O}(1)$ parameters can account for one or two units of charge differences. Here we choose to consider just two representative models that have been presented in Ref. [10] and more carefully analyzed in Ref. [11]. The first one, “Anarchy”, features degenerate charges of left-handed lepton doublets, so that all mixing angles are predicted to be $\mathcal{O}(1)$. The second one, “Hierarchy”, has non-degenerate charges in order to account for the relative smallness of $\theta _{13}$ and $\Delta m^2_\mathrm{solar}/\Delta m^2_\mathrm{atm}$. Other models that have been considered in Ref. [10, 39] fall in between these two models as regards their phenomenological consequences in FGM. The charge assignments of the two models are given by

- Anarchy
  23 $$\begin{aligned}&E_i = (3,2,0) \quad L_i = (L_3,L_3,L_3),\quad N_i = (0,0,0),\nonumber \\&\epsilon _A \approx 0.2. \end{aligned}$$
- Hierarchy
  24 $$\begin{aligned}&E_i = (5,3,0) \quad L_i = (2+L_3,1+L_3,L_3), \nonumber \\&N_i = (2,1,0),\quad \epsilon _H \approx 0.3. \end{aligned}$$

For simplicity the expansion parameters are taken here as the central values of the accurate fit in Ref. [11], although there is of course some range due to the unknown order one coefficients. We will use these values in the numerical analysis of Sect. 4. Note the dependence on an overall charge shift $L_3$ that essentially corresponds to $\tan \beta$.

### Application to FGM

We now discuss the implementation of the above $U(1)$ models in FGM. For this we only have to specify the charges of the messengers. While in principle they can be arbitrary, we only consider the simplest choice in which they have the same charges as the Higgs fields, i.e. they transform trivially under the flavor symmetry $\Phi = \overline{\Phi } = 0$. This immediately implies that the new couplings of matter fields to the messengers have exactly the same parametric suppression as the corresponding matter-Higgs couplings, but with different $\mathcal{O}(1)$ coefficients. In order to see this point more explicitly, we go to the mass basis for the charged leptons by means of the superfield transformations

25 $$\begin{aligned} L&= \begin{pmatrix} L_N \\ L_E \end{pmatrix} \rightarrow \begin{pmatrix} V_{NL} L_N \\ V_{EL} L_E \end{pmatrix}, \quad E \rightarrow V_{ER} E, \end{aligned}$$

so that

26 $$\begin{aligned} y_E&\rightarrow V_{EL}^T y_E V_{ER} = y_E^\mathrm{diag}. \end{aligned}$$

Note that in $U(1)$ models the rotations have the simple parametric structure

27 $$\begin{aligned} (V_{EL})_{ij} \sim \epsilon ^{|L_i - L_j|} \quad (V_{ER})_{ij} \sim \epsilon ^{|E_i - E_j|}. \end{aligned}$$

The spurion $\lambda _E$ transforms accordingly under the above rotations. However, it is straightforward to check that its parametric flavor suppression remains unchanged and only the $\mathcal{O}(1)$ coefficients do change so that $V_{EL}^T \lambda _E V_{ER} \sim \lambda _E$. As a result, since the flavor suppression of $\lambda _E$ is the same of $y_E$, one gets in the mass basis

28 $$\begin{aligned} (y_E)_{ii} = a_{ii} \epsilon ^{L_i + E_i}, \qquad (\lambda _E)_{ij} = \kappa _{ij} \epsilon ^{L_i + E_j}, \end{aligned}$$

where $a_{ii}$ and $\kappa _{ij}$ account for unknown, flavor dependent, $\mathcal{O}(1)$ coefficients. Assuming hierarchical charges ($E_3 \le E_{k}$ etc.), we finally find the following MIs:

29 $$\begin{aligned} (\delta _{LL}^e)_{ij}&\sim \epsilon ^{L_i + L_j + 2 E_3} \sim y^2_\tau \epsilon ^{L_i + L_j - 2 L_3},\end{aligned}$$

30 $$\begin{aligned} (\delta _{RR}^e)_{ij}&\sim \epsilon ^{E_i + E_j + 2 L_3} \sim y^2_\tau \epsilon ^{E_i + E_j - 2 E_3}, \end{aligned}$$

and similarly

31 $$\begin{aligned} (\delta _{LR}^e)_{ij} \sim y^2_\tau ~\frac{m^e_j~\epsilon ^{L_i + L_j - 2 L_3} + 2 m^e_i~\epsilon ^{E_i + E_j - 2 E_3}}{\sqrt{\tilde{m}_L \tilde{m}_E}}, \end{aligned}$$

where the overall coefficients are given by a calculable part that can be read off from Eqs. (13), (14) and an unknown $\mathcal{O}(1)$ coefficient coming from Eq. (28).

As already discussed, since the diagonal A-terms are real, the EDMs can only be generated by means of the combination of MIs $(\delta _{LL}^e)_{ik} (\delta _{LR}^e)_{kj}$, $(\delta _{LR}^e)_{ik} (\delta _{RR}^e)_{kj}$, and $(\delta _{LL}^e)_{ik} (\delta _{LR}^e)_{kk} (\delta _{RR}^e)_{kj}$ when $ij=11$. In particular, it turns out that the leading effect is captured by

32 $$\begin{aligned} (\delta _{LL}^e)_{i3} (\delta _{LR}^e)_{33} (\delta _{RR}^e)_{3j}&\sim \frac{\mu \tan \beta \, m_\tau }{\tilde{m}_L \tilde{m}_E } y_\tau ^3 \epsilon ^{L_i + E_j}, \end{aligned}$$

which involves additional powers of $y_\tau$. In principle, the effective MI of Eq. (32) also contributes to $\mu \rightarrow e\gamma$ when $ij=12,21$, however, within our models, single MI contributions always dominate.

**Fig. 1:**
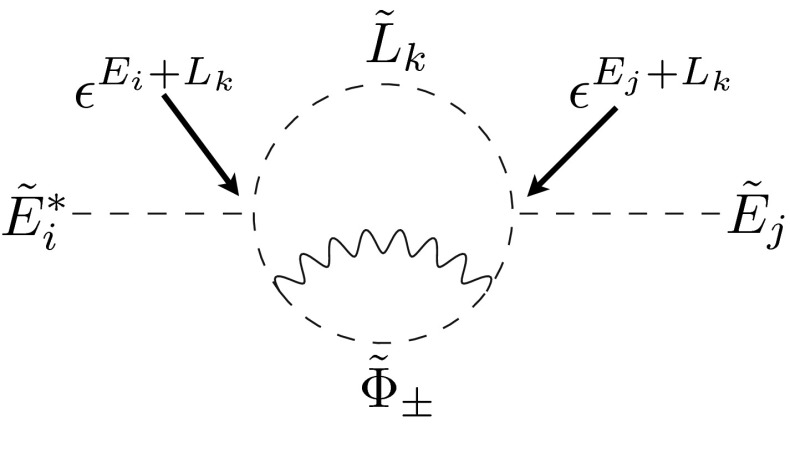
Example diagram for the 2-loop generation of $(\delta ^e_{RR})_{ij}$. $\tilde{L}_i,\tilde{E}_i$ denotes the scalar components of the superfields $L_i,E_i$ and $\tilde{\Phi }_\pm$ denotes the scalar mass eigenstates of the messengers

We conclude this section with a discussion of the general structure of the flavor suppression in these terms. First of all, note that LL and RR mass insertions are suppressed by powers of the spurion that are the *sum* of U(1) charges, in contrast to the leading-order terms allowed by the symmetry that have powers given by charge differences. The origin of this suppression is due to the fact that the $U(1)$ controls soft terms only indirectly via the messenger sector, which in turn generates soft terms only at loop level, thus leading to a double suppression by small couplings. As can be seen e.g. in Eq. (30) and the corresponding 2-loop diagram for $\delta ^e_{RR}$ in Fig. 1, this suppression can be split into two parts, one given by the sum of charges of the external sfermions and the second by (twice) the charge of the field that runs in the loop together with the messenger.

As we will discuss later on, the first suppression is exactly the same as in SUSY partial compositeness, while the second can lead to a further suppression by powers of $y_\tau$.

**Fig. 2:**
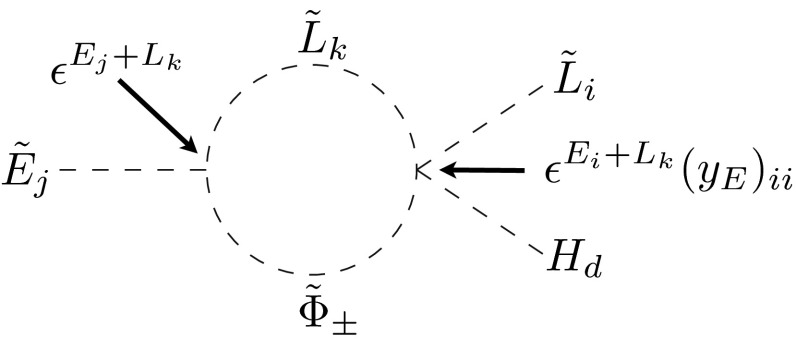
Example diagram for the 1-loop generation of $(\delta ^e_{LR})_{ij}$ in the fermion mass basis

Turning to LR mass insertions, again the loop origin implies a much stronger suppression than the leading-order term $\epsilon ^{L_i + E_j}$ respecting the $U(1)$ symmetry; see Fig. 2. This suppression is partially due to the alignment to Yukawas in the pure LR term, which potentially can be avoided in the effective LR terms at the price of an additional suppression by powers of $y_\tau$. This is also the only way in which phases can arise in the diagonal elements, since the pure LR term is always the product of a hermitian and a real diagonal matrix.

## Flavor phenomenology

We are now ready to discuss the lepton flavor phenomenology of the FGM model, which includes LFV processes with an underlying $\mu \rightarrow e$ transition, the electron EDM $d_e$ and the anomalous magnetic moment of the muon $a_\mu \equiv (g-2)/2$. The current experimental bounds and future sensitivities for some of the most relevant LFV channels and for $d_e$ are reported in Table 1. On the other hand, $a_\mu$ currently shows a discrepancy between the SM prediction and the experimental value [50–57]

33 $$\begin{aligned} \Delta a_\mu = a_\mu ^\mathrm{EXP}-a_\mu ^\mathrm{SM} = 2.90 (90) \times 10^{-9}. \end{aligned}$$

One of the goals of the present paper is to investigate whether it is possible to explain this anomaly in our model while being compatible with LFV and EDM bounds.

**Table 1:** Current experimental bounds and future sensitivities for some low-energy LFV observables and the electron EDM

LFV process	Present bound	Future sensitivity
$\mu \rightarrow e \gamma$	$5.7 \times 10^{-13}$ [41]	$\approx 6 \times 10^{-14}$ [42]
$\mu \rightarrow 3 e$	$1.0 \times 10^{-12}$ [43]	$\approx 10^{-16}$ [44]
$\mu ^-$ Au $\rightarrow$ $e^-$ Au	$7.0 \times 10^{-13}$ [45]	$?$
$\mu ^-$ Ti $\rightarrow$ $e^-$ Ti	$4.3 \times 10^{-12}$ [46]	$?$
$\mu ^-$ Al $\rightarrow$ $e^-$ Al	$-$	$\approx 10^{-16}$ [47, 48]
Electron EDM	Present bound	Future sensitivity
$d_e (\mathrm{e~cm})$	$8.7 \times 10^{-29}$ [49]	$?$

Concerning LFV processes, hereafter we focus only on $\mu \rightarrow e \gamma$ since it represents the best probe of our scenario. The branching ratio of $\mu \rightarrow e \gamma$ is defined as

34 $$\begin{aligned} \mathrm{BR}(\mu \rightarrow e \gamma ) = \frac{48\pi ^3 \alpha }{G^2_F} \left( \left| A^{21}_L\right| ^2 + \left| A^{21}_R\right| ^2 \right) , \end{aligned}$$

where the amplitudes $A^{21}_L$ and $A^{21}_R$, in the limit of $M_1 = M_2 = \mu = \tilde{m}_L = \tilde{m}_R = \tilde{m}$ and keeping only $\tan \beta$ enhanced terms,^6^ read

35 $$\begin{aligned} A^{21}_L&= \frac{4\alpha _2 + 5\alpha _Y}{240\pi }~\frac{\tan \beta }{{\tilde{m}}^2}~(\delta _{LL}^e)_{21}\nonumber \\&+ \frac{\alpha _Y}{48\pi }\left( \frac{{\tilde{m}}}{m_\mu }\right) ~\frac{1}{{\tilde{m}}^2}~(\delta _{LR}^e)^{*}_{12},\end{aligned}$$

36 $$\begin{aligned} A^{21}_R&= -\frac{\alpha _Y}{240\pi }~\frac{\tan \beta }{{\tilde{m}}^2}~(\delta _{RR}^e)_{21} + \frac{\alpha _Y}{48\pi }\left( \frac{{\tilde{m}}}{m_\mu }\right) ~\frac{1}{{\tilde{m}}^2}~(\delta _{LR}^e)_{21}.\nonumber \\ \end{aligned}$$

Notice that in the above amplitudes we have kept only single MI effects since they are dominant in our scenarios. The expressions for $\Delta a_\mu$ and $d_e$ are well approximated by

37 $$\begin{aligned}&\Delta a_\mu = \frac{5\alpha _2 + \alpha _Y}{48\pi }\frac{m^2_\mu }{{\tilde{m}}^2}\tan \beta ,\end{aligned}$$

38 $$\begin{aligned}&\frac{d_e}{e} = \frac{\alpha _Y}{120\pi }\frac{m_\tau }{{\tilde{m}}^2}\tan \beta ~ \mathrm{Im} [(\delta _{LL}^e)_{13} (\delta _{RR}^e)_{31}]. \end{aligned}$$

In order to highlight the relevant effects, we now provide some numerical estimates for the above observables outlining also their possible correlations. We find that

39 $$\begin{aligned} \mathrm{BR}(\mu \rightarrow e \gamma )&\approx 3 \times 10^{-14}\left( \frac{200~\mathrm{GeV}}{{\tilde{m}}}\right) ^4\nonumber \\&\tan ^2\beta ~\left( \frac{|(\delta _{LL}^e)_{21}|}{10^{-4}}\right) ^2,\end{aligned}$$

40 $$\begin{aligned} \Delta a_\mu&\approx 3 \times 10^{-10} \left( \frac{200~\mathrm{GeV}}{{\tilde{m}}}\right) ^2\tan \beta , \end{aligned}$$

41 $$\begin{aligned} |d_e|&\approx 2 \times 10^{-29} \left( \frac{200~\mathrm{GeV}}{{\tilde{m}}}\right) ^2 \nonumber \\&\tan \beta ~ \left| \frac{\mathrm{Im} [(\delta _{LL}^e)_{13} (\delta _{RR}^e)_{31}]}{10^{-6}}\right| e\mathrm{~cm}. \end{aligned}$$

Making the correlations among BR$(\mu \rightarrow e \gamma )$, $\Delta a_\mu$ and $d_e$ more explicit, it turns out that

42 $$\begin{aligned}&\mathrm{BR}(\mu \rightarrow e \gamma ) \approx 3 \times 10^{-13}\left( \frac{\Delta a_\mu }{10^{-9}}\right) ^2 \left( \frac{|(\delta _{LL}^e)_{21}|}{10^{-4}}\right) ^2,\end{aligned}$$

43 $$\begin{aligned}&|d_e| \approx 7 \times 10^{-29} \left( \frac{\Delta a_\mu }{10^{-9}}\right) \left| \frac{\mathrm{Im} [(\delta _{LL}^e)_{13} (\delta _{RR}^e)_{31}]}{10^{-6}}\right| e\mathrm{~cm}.\nonumber \\ \end{aligned}$$

Equations (39–40) deserve a few comments:

- In both flavor models we have considered, the dominant contribution to BR$(\mu \rightarrow e\gamma )$ stems from $A^{21}_L$, in particular from the $\tan \beta$-enhanced term proportional to $(\delta _{LL}^e)_{21}$, due to smaller flavor hierarchies in the left-handed lepton sector.
- The dominant $\mu \rightarrow e\gamma$ amplitude grows with $\tan \beta$ as $A^{21}_L \sim (\delta _{LL}^e)_{21} \tan \beta \sim \tan ^3\beta$, since $(\delta _{LL}^e)_{21} \sim y^2_\tau \approx 10^{-4}\tan ^2\beta$, which implies that $A^{21}_L$ is very efficiently suppressed for relatively low $\tan \beta$. As a result, the very stringent experimental bound on BR$(\mu \rightarrow e\gamma )$ might be fulfilled even for a light spectrum ${\tilde{m}}\sim 200~$GeV provided $\tan \beta \sim 1$.
- The electron EDM can be induced at the leading order only through the effective MI of Eq. (32) and it turns out that $d_e \sim \tan ^5\beta$. Therefore $d_e$ is well under control for low $\tan \beta$ values, analogously to BR$(\mu \rightarrow e\gamma )$.
- The $a_\mu$ anomaly can be accounted for while satisfying the stringent bounds from BR$(\mu \rightarrow e \gamma )$ and $d_e$, only provided that the relevant flavor mixing angles are suppressed at the level of $(\delta _{LL}^e)_{21} \lesssim 10^{-4}$ and $(\delta _{LL}^e)_{13} (\delta _{RR}^e)_{31} \lesssim 10^{-6}$.

In order to quantify the above considerations, we specialize now to the $U(1)$ flavor models that have been introduced in the previous section: the *anarchical* and the *hierarchical* models. The predictions of other scenarios discussed in Refs. [10, 11], fall in between the ones we discuss here. In these two models, the relevant MIs entering the predictions of BR$(\mu \rightarrow e \gamma )$ and $d_e$ are estimated as:

- Anarchy
  44 $$\begin{aligned} (\delta _{LL}^e)_{21}&\approx \kappa \frac{6}{N}~y^2_\tau \approx \kappa \frac{6 \times 10^{-4}}{N}\tan ^2\beta , \nonumber \\ (\delta _{LL}^e)_{13} (\delta _{RR}^e)_{31}&\approx \kappa ' \frac{200}{N^2}~y^4_\tau \epsilon _A^{3} \nonumber \\&\approx \kappa ' \frac{2 \times 10^{-8}}{N^2} \left( \frac{\epsilon _A}{0.2}\right) ^3\tan ^4\beta . \end{aligned}$$
- Hierarchy
  45 $$\begin{aligned} (\delta _{LL}^e)_{21}&\approx \kappa \frac{6}{N}~y^2_\tau \epsilon ^{3}_H \approx \kappa \frac{2 \times 10^{-5}}{N}\tan ^2\beta \left( \frac{\epsilon _H}{0.3}\right) ^3, \nonumber \\ (\delta _{LL}^e)_{13} (\delta _{RR}^e)_{31}&\approx \kappa ' \frac{200}{N^2}~y^4_\tau \epsilon _H^{7}\nonumber \\&\approx \kappa ' \frac{4 \times 10^{-10}}{N^2} \left( \frac{\epsilon _H}{0.3}\right) ^7\tan ^4\beta . \end{aligned}$$

where we have used Eqs. (13), (28) assuming an intermediate mediation scale $M \sim 10^{10} \, \mathrm{GeV}$. Moreover, we have explicitly included the dependence on the unknown $\mathcal{O}(1)$ coefficients parameterized through $\kappa$ and $\kappa '$ that are defined as

46 $$\begin{aligned} \kappa&\equiv \frac{\kappa _{23} \kappa _{13}^*}{a_{33}^2}, \quad \kappa ' \equiv \frac{\kappa _{13} \kappa _{31} \kappa _{33}^{*2} }{a_{33}^4}. \end{aligned}$$

A prominent feature emerging from Eqs. (44)–(45) is the sensitivity of the MIs to the number of messenger $N$, since the diagonal sfermion masses are dominated by the MGM contribution proportional to $N$. We finally get for the $\mu \rightarrow e \gamma$ branching ratio

47 $$\begin{aligned} \mathrm{BR}(\mu \rightarrow e \gamma )&\approx \tan ^6\beta \left( \frac{\kappa }{N} \right) ^2 \left( \frac{200~\mathrm{GeV}}{{\tilde{m}}}\right) ^4\nonumber \\&\quad \times {\left\{ \begin{array}{ll} 1 \times 10^{-12} &{} \mathrm{Anarchy} \\ 9 \times 10^{-16} &{} \mathrm{Hierarchy} \end{array}\right. } \end{aligned}$$

48 $$\begin{aligned}&\approx \tan ^4\beta \left( \frac{\kappa }{N} \right) ^2 \left( \frac{\Delta a_\mu }{10^{-9}}\right) ^2 \nonumber \\&\quad \times {\left\{ \begin{array}{ll} 1 \times 10^{-11} &{} \mathrm{Anarchy} \\ 9 \times 10^{-15} &{} \mathrm{Hierarchy} \end{array}\right. } \end{aligned}$$

and the eEDM

49 $$\begin{aligned} |d_e|&\approx \tan ^5\beta \left( \frac{\kappa '}{N^2} \right) \left( \frac{200~\mathrm{GeV}}{{\tilde{m}}}\right) ^2 e\mathrm{~cm}\nonumber \\&\quad \times {\left\{ \begin{array}{ll} 4 \times 10^{-31} &{} \mathrm{Anarchy} \\ 1 \times 10^{-32} &{} \mathrm{Hierarchy} \end{array}\right. } \end{aligned}$$

50 $$\begin{aligned}&\approx \tan ^4\!\beta \! \left( \frac{\kappa '}{N^2} \right) \! \left( \!\frac{\Delta a_\mu }{10^{-9}}\!\right) e\mathrm{~cm}\nonumber \\&\quad \times {\left\{ \begin{array}{ll} 1 \times 10^{-30} &{} \mathrm{Anarchy} \\ 3 \times 10^{-32} &{} \mathrm{Hierarchy}. \end{array}\right. } \end{aligned}$$

Reformulating the constraint from $\mu \rightarrow e \gamma$ as a bound on the SUSY scale gives approximately

51 $$\begin{aligned} \frac{\tilde{m}}{200 \, \mathrm{GeV}}&\gtrsim \tan ^{3/2} \beta \sqrt{\frac{\kappa }{N} } \left( \frac{\mathrm{BR}(\mu \rightarrow e \gamma ) }{5.7 \times 10^{-13}} \right) ^{-1/4} \nonumber \\&\qquad \times {\left\{ \begin{array}{ll} 1 &{} \mathrm{Anarchy} \\ 0.2 &{} \mathrm{Hierarchy}. \end{array}\right. } \end{aligned}$$

Having outlined the expected behaviors and main features of flavor observables within our FGM setup supplemented by $U(1)$ flavor models, we are ready now to perform a complete numerical analysis.

**Fig. 3:**
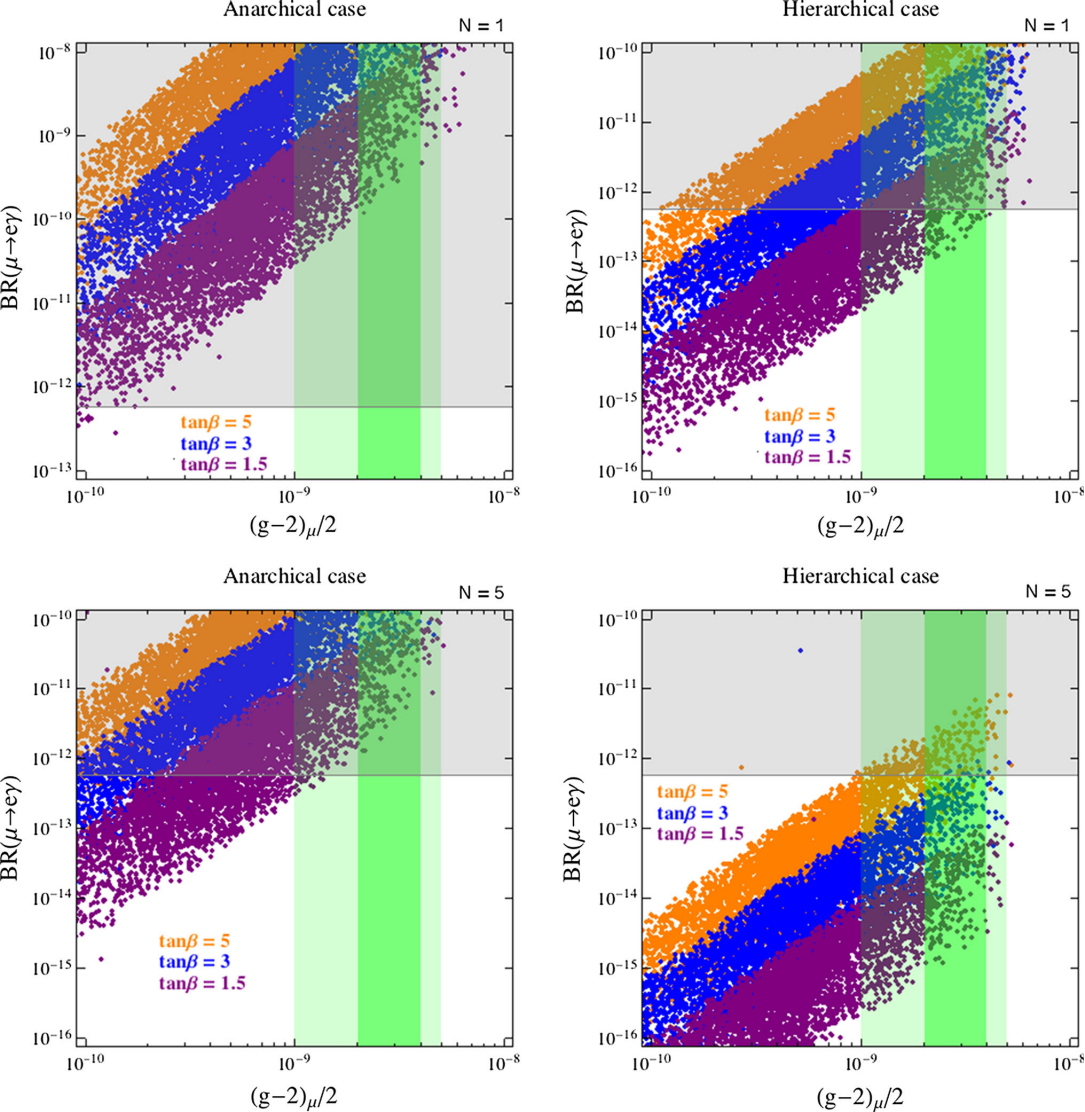
Predictions for BR$(\mu \rightarrow e\gamma )$ vs. $\Delta a_\mu$ for different values of $\tan \beta$: *purple*, *blue* and *orange dots* correspond to $\tan \beta = 1.5, 3, 5$, respectively. The plots on the *left* (*right*) refer to the anarchical (hierarchical) case. For the *upper* (*lower*) plots the number of messenger is set to $N = 1~(5)$. *Gray regions* are excluded by MEG [41]. In the *green* (*dark green*) bands the $(g-2)_\mu$ discrepancy is reduced below the 2$\sigma$ (1$\sigma$) level

**Fig. 4:**
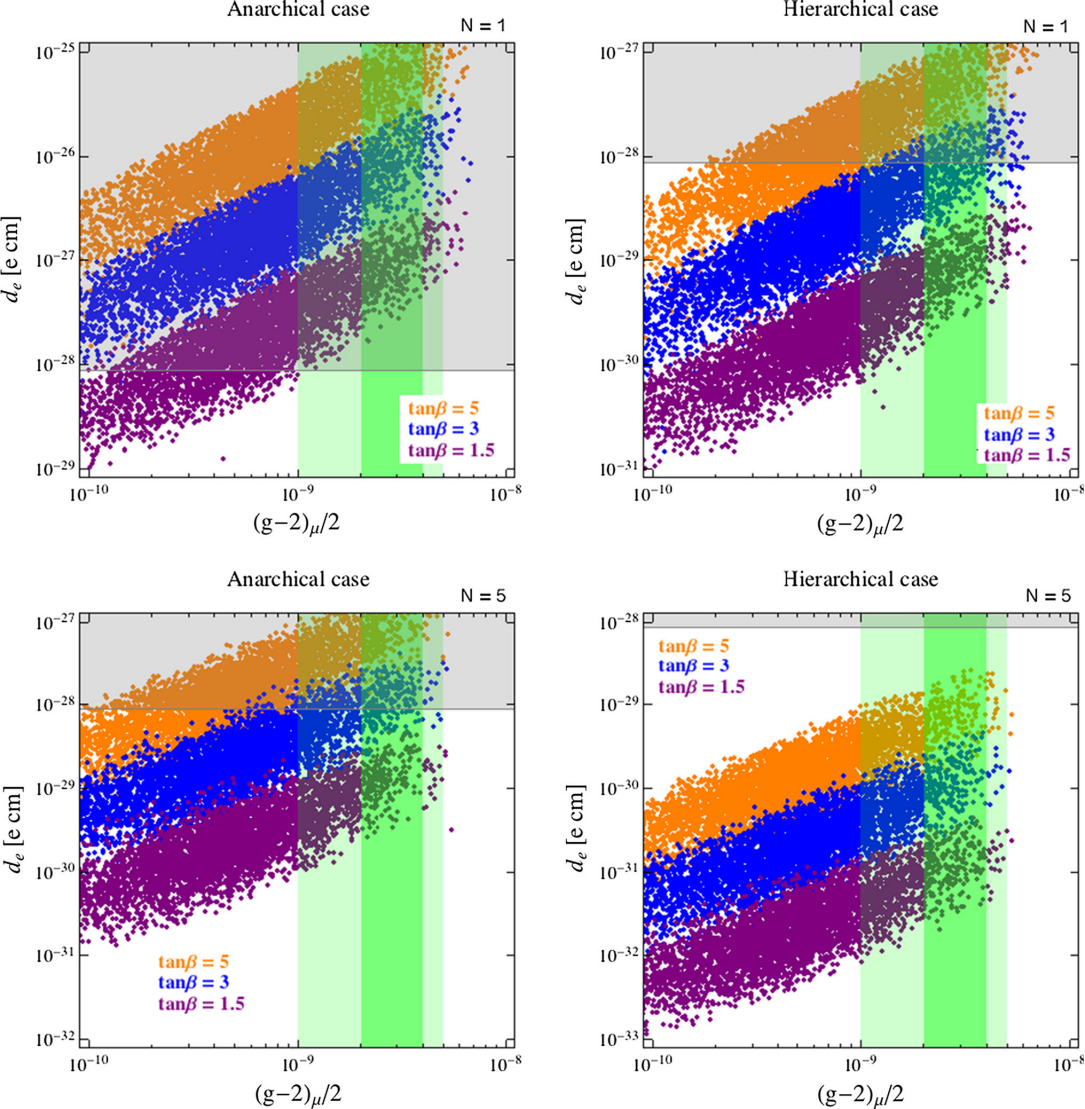
The same scenarios as in Fig. 3 for $d_e$ vs. $\Delta a_\mu$. *Gray regions* are excluded by ACME [49]. In the *green* (*dark green*) bands the $(g-2)_\mu$ discrepancy is reduced below the 2$\sigma$ (1$\sigma$) level

In Fig. 3, we show the predictions for BR$(\mu \rightarrow e\gamma )$ vs. $\Delta a_\mu$ for different values of $\tan \beta$: purple, blue and orange dots correspond to $\tan \beta = 1.5, 3, 5$, respectively. The plots on the left (right) refer to the anarchical (hierarchical) case. For the upper (lower) plots the number of messenger is set to $N = 1~(5)$. In Fig. 4, we show the analogous plots for $d_e$ vs. $\Delta a_\mu$. In the scan we have varied the unknown $\mathcal {O}(1)$ coefficients $\kappa , \kappa '$ for the MIs in the range $(0.3, 1.5)$. The other parameters were varied in the following ranges:

52 $$\begin{aligned}&10^6 ~\mathrm{GeV}\le M \le 10^{15} ~\mathrm{GeV},~~ 100~\mathrm{GeV} \!\le \! \tilde{m}_{E}(M) \!\le \! 1~\mathrm{TeV},\nonumber \\&\quad 100~\mathrm{GeV} \le \mu \le \mu _\mathrm{max}, \end{aligned}$$

where $\mu _\mathrm{max} \equiv \tilde{m}_{\tau _L} \tilde{m}_{\tau _R}/(m_\tau \tan \beta )$ is the maximal value giving a non-tachyonic stau. Low-energy values of slepton and gaugino masses were obtained by solving the 1-loop renormalization group equations.

In the plots, the gray shaded regions are excluded by the current bounds from $\mu \rightarrow e\gamma$ or $d_e$ reported in Table 1, while the green (dark green) area approximately corresponds to values of $\Delta a_\mu$ lowering the discrepancy below the 2$\sigma$ (1$\sigma$) level.

**Fig. 5:**
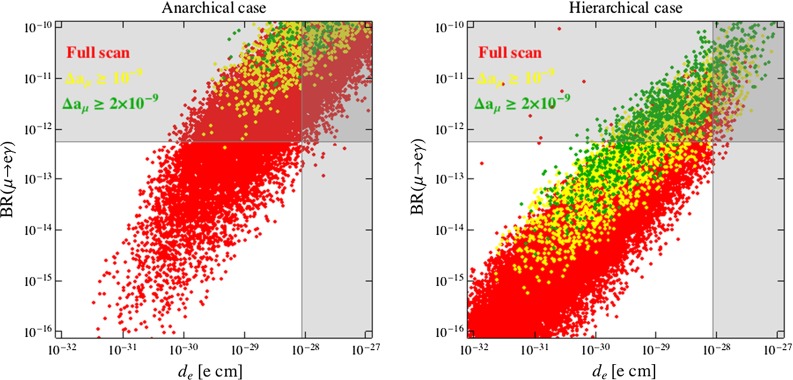
Predictions for BR$(\mu \rightarrow e\gamma )$ vs. $d_e$ for the anarchical (*left*) and hierarchical (*right*) cases. *Yellow* (*green*) points correspond to $\Delta a_\mu \ge 10^{-9}$ ($2\times 10^{-9}$). *Gray regions* are excluded by MEG [41] and/or ACME [49]

A direct comparison of the bounds and the discovery potential of $\mu \rightarrow e\gamma$ and $d_e$ is shown in Fig. 5, where we plot the result of a random variation of the full set of parameters for the anarchical (left) and hierarchical (right) cases:

53 $$\begin{aligned}&10^6 ~\mathrm{GeV}\le M \le 10^{15} ~\mathrm{GeV},~~ 100~\mathrm{GeV} \le \tilde{m}_{E}(M) \le 2~\mathrm{TeV},\nonumber \\&\quad 100~\mathrm{GeV} \le \mu \le \mu _\mathrm{max}, \nonumber \\&1 \le N \le 5,\quad 1.5 \le \tan \beta \le 5,\quad 0.3\le \kappa , \kappa ^\prime \le 1.5. \end{aligned}$$

In addition, the yellow (green) points correspond to $\Delta a_\mu \ge 10^{-9}$ ($2\times 10^{-9}$).

The main results emerging from our numerical analysis can be summarized as follows:

- In the anarchical scenario, it is very difficult if not impossible to explain the $\Delta a_\mu$ anomaly because of the strong bounds from both BR$(\mu \rightarrow e\gamma )$ and $d_e$ (see left panels of Figs. 3–5). The latter observables have a comparable sensitivity to the scenario in question and might reach experimentally visible values even for SUSY masses far beyond the LHC reach in the multi-TeV regime. As already discussed, BR$(\mu \rightarrow e\gamma )$ and $d_e$ grow fast with $\tan \beta$ (like $\tan ^6\beta$ and $\tan ^5\beta$, respectively) and are both suppressed by increasing $N$. As a result, the scenario with low $\tan \beta$ and $N=5$ (we remind the reader that for low mediation scales perturbativity requires $N \lesssim 5$) is the most favorable scenario, as clearly shown in Figs. 3 and 4.
- The hierarchical scenario easily offers the possibility to explain the $\Delta a_\mu$ anomaly while satisfying the limits on BR$(\mu \rightarrow e\gamma )$ and $d_e$ (right panels of Figs. 3, 4, 5). This happens thanks to the stronger suppression of the flavor mixing angles compared to the anarchical case. On the other hand, all the other considerations made above for the anarchical case apply here as well.
- $\mu \rightarrow e\gamma$ and $d_e$ have comparable sensitivities, but $\mu \rightarrow e\gamma$ is currently more constraining, as we can see from Fig. 5. Interestingly, an improvement of the sensitivity by one or two orders of magnitude would make the electron EDM the most powerful probe of FGM scenarios especially in the case of heavy superpartners, corresponding to the red points in Fig. 5. This is a consequence of the slower decoupling of $d_e$ with respect to the NP scale: $d_e \sim {\tilde{m}}^{-2}$, while BR$(\mu \rightarrow e\gamma )\sim {\tilde{m}}^{-4}$.
- Given the expected future sensitivities to the $\mu \rightarrow e$ transitions reported in Table 1 and the following approximate relations among different modes (branching ratios of $\mu \rightarrow e\gamma$ and $\mu \rightarrow eee$, $\mu \rightarrow e$ conversion rate (CR) in nuclei):
  54 $$\begin{aligned}&{\mathrm{BR}(\mu \rightarrow eee)} \simeq \frac{\alpha }{3\pi } \bigg (\log \frac{m^2_{\mu }}{m^2_{e}}-3\bigg ) \mathrm{BR}(\mu \rightarrow e\gamma ), \nonumber \\&\mathrm{CR}(\mu \rightarrow e~\text{ in } \text{ N }) \simeq \alpha \times \mathrm{BR}(\mu \rightarrow e\gamma ), \end{aligned}$$
  we see that there are good prospects for a full test of the parameter space favored by $\Delta a_\mu$ at future experiments.

Let us now also show how the $\mu \rightarrow e\gamma$ and $d_e$ constraints appear in terms of the gaugino and slepton masses. For illustration purposes, we adopt a more general low-energy spectrum than the one predicted by MGM, which allows us to parameterize in a model-independent way possible distortions of the spectrum due to the full set of matter-messenger couplings studied in [25], including the other couplings in Eq. (4), as well as more generic SUSY breaking sectors, in the spirit of General Gauge Mediation [58]. In practice, we still use Eqs. (9–11) to set the off-diagonal entries but we treat slepton and gaugino masses as free parameters at low energy.

**Fig. 6:**
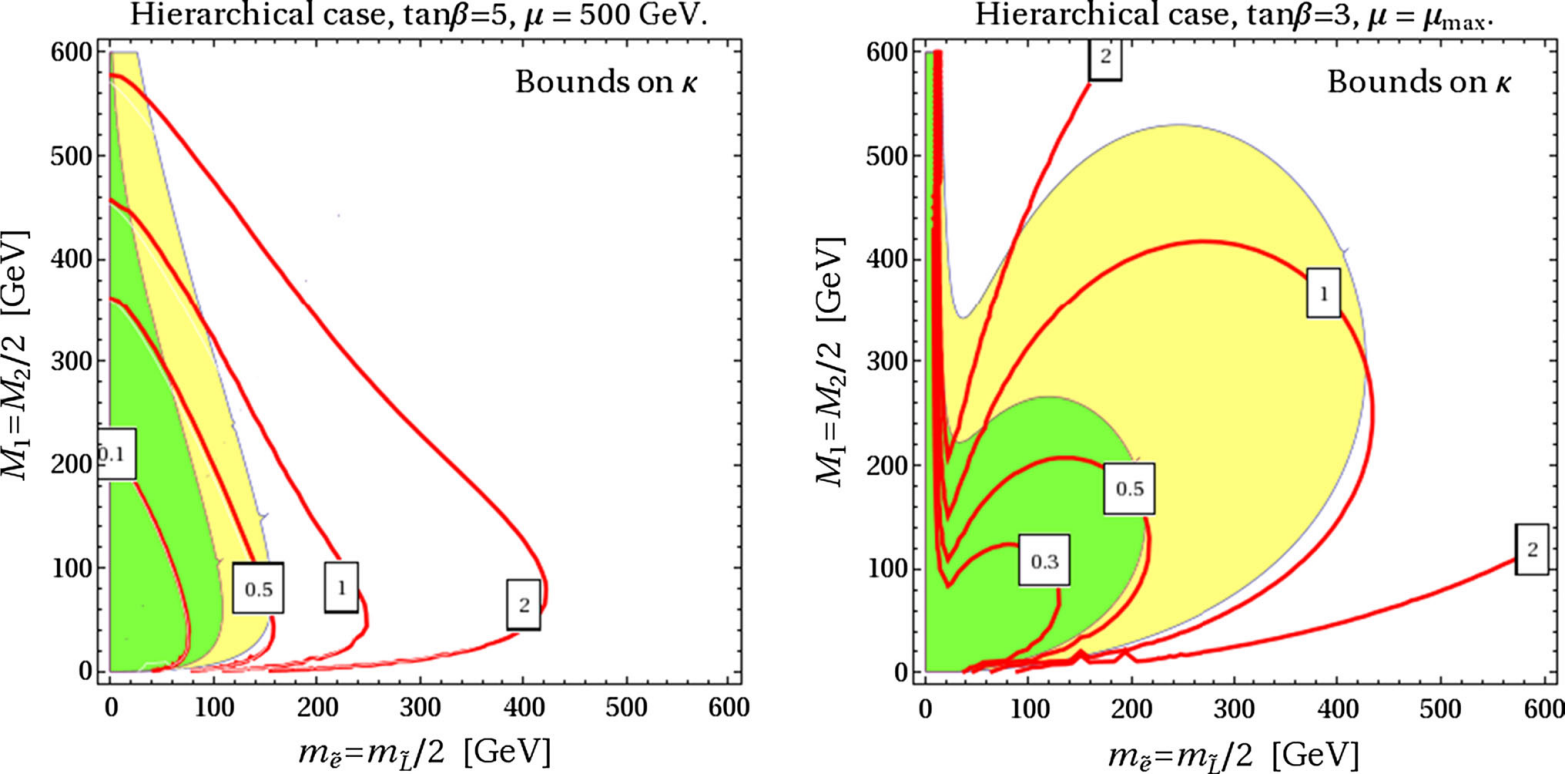
Bounds on the $\mathcal {O}(1)$ coefficient $\kappa$, see Eq. (46), from $\mu \rightarrow e\gamma$ in the hierarchical scenario. For definiteness, we have assumed $M_2 = 2 M_1$, $\tilde{m}_L = 2 \tilde{m}_E$ and different choices of $\mu$ and $\tan \beta$. The *yellow* (*green*) areas give $\Delta a_\mu \ge 10^{-9}$ ($2\times 10^{-9}$)

In Fig. 6, we show the current bounds on the $\mathcal {O}(1)$ coefficient $\kappa$, as defined in Eq. (46), from $\mu \rightarrow e\gamma$ for different choices of $\mu$ and $\tan \beta$ in the hierarchical case. For definiteness, we fixed the relation among gauginos and slepton masses as follows: $M_2 = 2\times M_1$, $\tilde{m}_L = 2\times \tilde{m}_E$. The yellow (green) areas give $\Delta a_\mu \ge 10^{-9}$ ($2\times 10^{-9}$). As we can see, it is not necessary that the unknown coefficients conspire to provide an unnaturally small suppression, in order to take $\mu \rightarrow e\gamma$ under control in the region favored by $(g-2)_\mu$.

**Fig. 7:**
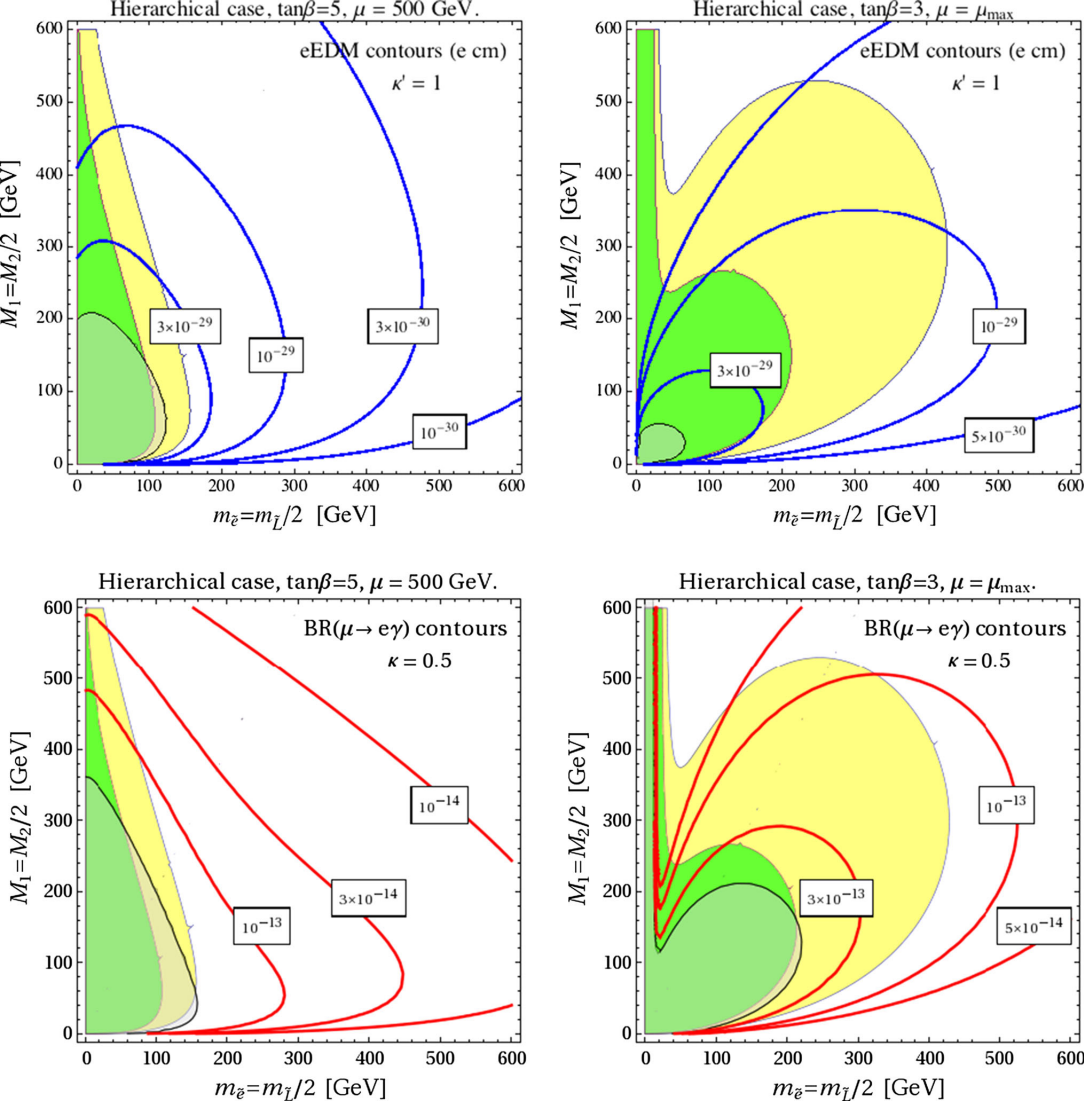
Contours of BR($\mu \rightarrow e\gamma$) and $d_e$ for the same choice of the parameters as in Fig. 6 and $\kappa =0.5$, $\kappa '=1$. The *gray shaded* regions are presently excluded by $\mu \rightarrow e\gamma$ or $d_e$. The *yellow* (*green*) areas give $\Delta a_\mu \ge 10^{-9}$ ($2\times 10^{-9}$)

In Fig. 7, we show contours of BR($\mu \rightarrow e\gamma$) and $d_e$ for the same choice of the parameters as above and the specific values $\kappa =0.5$, $\kappa '=1$, in order to illustrate the present bounds and the possible impact of the future experiments in terms of the masses of the SUSY particles in the game. In particular, the gray shaded regions are presently excluded by $\mu \rightarrow e\gamma$ or $d_e$. Again, we see that a large contribution to $(g-2)_\mu$ is perfectly compatible with the present bounds, but there are good prospects for a full test of the relevant parameter space in the future.

Besides the LEP constraints (corresponding to $\tilde{m}_E,~\tilde{m}_L, M_2\gtrsim 100~\mathrm{GeV}$), the mass plane shown in the above plots is now challenged by searches for the electroweak production of SUSY particles performed by the LHC experiments, based on events with two or more leptons plus missing transverse momentum. The exact bounds are model dependent and their precise derivation is beyond the scope of the present study. Nevertheless, we briefly summarize here their possible impact.

Since we are considering scenarios with gauge-mediated SUSY breaking, the LSP is always a practically massless gravitino. The limits set by LHC searches then strongly depend on the nature and the life-time of the next to LSP (NLSP). In case the sleptons are lighter than the Bino, as it can occur even in MGM for large values of $N$, each decay chain would end with the degenerate sleptons NLSP decaying into leptons and gravitino. If this decay occurs promptly (which requires low mediation scales, $M\lesssim 10^6$ GeV), then the bounds from direct (Drell–Yan) slepton production translate to a limit on the mass of the right-handed (left-handed) sleptons at about 250 (300) GeV [59]. In case sleptons are long-lived compared to the detector scale, searches for charged tracks set a bound on degenerate slepton NLSP mass at about 400 GeV [60]. Interestingly, no searches performed so far constrain the intermediate case, featuring a disappearing track with a displaced vertex inside the detector, occurring for a wide range of the messenger scale, $10^6~\mathrm{GeV} \lesssim M \lesssim 10^9~\mathrm{GeV}$ [61]. The above limits can be substantially relaxed if the Bino is lighter than the sleptons and escapes the detector, thus resembling searches within gravity mediation. In particular for a neutralino NLSP heavier than about 150 GeV, there is no constraint from direct slepton production [59].

The most stringent constraint would occur in the case of the hierarchy $M_1 < \tilde{m}_L < M_2$ from Wino-like chargino/neutralino production followed by decays into on-shell sleptons/sneutrinos, with bounds up to 700 GeV on the Wino mass from multi-lepton plus missing energy searches [62]. However, such searches lose sensitivity if the mass splitting of the sleptons with either the Bino or the Wino gets small.

Comparing the limits reported above, with our plots in Fig. 6 and 7, we see that there is still room for a large SUSY contribution to $a_\mu$, at least at the $10^{-9}$ level (yellow regions), especially if $\mu$ approaches the maximal value $\mu _\mathrm{max} \equiv \tilde{m}_{\tau _L} \tilde{m}_{\tau _R}/(m_\tau \tan \beta )$. This conclusion is supported by the results of Refs. [63, 64] where a systematic study of the LHC constraints on the parameter space favored by $(g-2)_\mu$ has been presented.

The LHC collaborations have obtained the limits discussed above from events with leptons and missing energy under the implicit assumption of no flavor mixing among the sleptons. This is the reason why they only employed flavor-conserving categories, e.g. opposite-sign, same-flavor leptons ($e^+e^-$ and $\mu ^+\mu ^-$) for the search of Drell–Yan slepton production [59]. The interpretation of these searches in terms of mass limits can, however, be affected in presence of large flavor mixing. For instance, di-slepton production can lead in such a case to a sizeable number of $e^\pm \mu ^\mp$ events, effectively relaxing the bounds on smuon/selectron masses obtained from $e^+e^-$ and $\mu ^+\mu ^-$ events alone. A quantitative discussion of this effect will be given elsewhere.

## Comparison with other models

In this section, we compare the peculiar flavor structure of FGM to other models that predict the parametric flavor suppression of soft terms. In particular, we consider $U(1)$ flavor models within SUGRA scenarios and models with SUSY partial compositeness (PC).

In those models the SUSY mediation scale $\Lambda _S$ is assumed to be above the scale of flavor messengers $\Lambda _F$, so that the flavor structure of soft terms at the scale $\Lambda _F$ is controlled entirely by the flavor dynamics at this scale, irrespectively of their structure at the scale $\Lambda _S$. In FGM the situation is reversed as the SUSY messenger scale $\Lambda _S = M$ is below $\Lambda _F$. We stress that this setup is therefore complementary to the other scenarios, allowing also for very low SUSY mediation scales. All the unspecified dynamics of the flavor sector is imprinted in the matter-messenger couplings, just like Yukawas, and the full SUSY spectrum is totally calculable in terms of these couplings.

### $U(1)$ and gravity mediation

In gravity mediation the natural expectation for soft terms at the flavor scale is given by the most general terms invariant under the symmetry using the flavon as a spurion. This gives for slepton mass insertions

55 $$\begin{aligned}&(\delta _{LR}^e)_{ij} \sim \frac{A v_d}{\tilde{m}_L \tilde{m}_E } \epsilon ^{L_i + E_j}, \end{aligned}$$

56 $$\begin{aligned}&(\delta _{LL}^e)_{ij} \sim \epsilon ^{|L_i - L_j |}, \quad (\delta _{RR}^e)_{ij} \sim \epsilon ^{|E_i - E_j |}. \end{aligned}$$

Focusing on the *anarchical* and *hierarchical* models of Sect. 3.1, the relevant MIs for BR$(\mu \rightarrow e\gamma )$ and $d_e$ are again $(\delta _{LL}^e)_{21}$ and $(\delta _{LL}^e)_{13}(\delta _{RR}^e)_{31}$, respectively. In the anarchic case we find:

- Anarchy
  57 $$\begin{aligned}&(\delta _{LL}^e)_{21} \sim 1,\qquad (\delta _{LL}^e)_{13}(\delta _{RR}^e)_{31} \sim \epsilon ^3_A, \end{aligned}$$
  leading to the following predictions:
  58 $$\begin{aligned}&\mathrm{BR}(\mu \rightarrow e \gamma ) \sim 5 \times 10^{-13}\left( \frac{10~\mathrm{TeV}}{{\tilde{m}}}\right) ^4 \tan ^2\beta ,\end{aligned}$$
  59 $$\begin{aligned}&|d_e| \sim 7 \times 10^{-29} \left( \frac{10~\mathrm{TeV}}{{\tilde{m}}}\right) ^2 \tan \beta \, e \mathrm{\, cm}. \end{aligned}$$
- Hierarchy
  60 $$\begin{aligned}&(\delta _{LL}^e)_{21} \sim \epsilon _H \sim 0.3,\qquad (\delta _{LL}^e)_{12}(\delta _{RR}^e)_{21} \sim \epsilon ^3_H, \end{aligned}$$
  where we took $(\delta _{LL}^e)_{12}(\delta _{RR}^e)_{21}$ instead of $(\delta _{LL}^e)_{13}(\delta _{RR}^e)_{31}$ since the contribution of the latter to the eEDM is smaller by a factor of $(y_{\tau }/y_{\mu })\times \epsilon ^{4}_{H} \sim 0.1$ compared to that induced by $(\delta _{LL}^e)_{12}(\delta _{RR}^e)_{21}$. We therefore have the following predictions:
  61 $$\begin{aligned}&\mathrm{BR}(\mu \rightarrow e \gamma ) \sim 7 \times 10^{-13}\left( \frac{5~\mathrm{TeV}}{{\tilde{m}}}\right) ^4 \tan ^2\beta ~\left( \frac{\epsilon _H}{0.3}\right) ^2, \end{aligned}$$
  62 $$\begin{aligned}&|d_e| \sim 6 \times 10^{-29} \left( \frac{5~\mathrm{TeV}}{{\tilde{m}}}\right) ^2 \tan \beta \, e \mathrm{\, cm}. \end{aligned}$$

As a result, single $U(1)$ flavor models with gravity mediation need sleptons well above the TeV scale, ${\tilde{m}} \gtrsim 10~\mathrm{TeV}\times \sqrt{\tan \beta } ~(5~\mathrm{TeV}\times \sqrt{\tan \beta })$ in the anarchical (hierarchical) scenario. Note that the bounds on the SUSY spectrum in the quark sector, in particular from $\epsilon _K$, are much stronger [28].

### SUSY partial compositeness

According to the paradigm of partial compositeness, the lepton Yukawa matrices have the form

63 $$\begin{aligned} (y_E)_{ij} \sim g_{\rho } \epsilon ^\ell _i \epsilon ^e_j, \end{aligned}$$

where $g_\rho$ is a strong coupling and $\epsilon ^{\ell ,e}_i \lesssim 1$ measures the amount of compositeness for the leptons. Such a scheme closely resembles the case of a single $U(1)$ flavor model, with the correspondence (in the limit of $g_\rho = 1$)

64 $$\begin{aligned} \epsilon ^{\ell ,e} _i \, \longleftrightarrow \, \epsilon ^{L_i,E_i}. \end{aligned}$$

As a result, the MIs are expected to take the following form [34]:

65 $$\begin{aligned} (\delta _{LL}^e)_{ij}&\sim \epsilon ^\ell _i \epsilon ^\ell _j \sim \epsilon ^{L_i + L_j }, \qquad (\delta _{RR}^e)_{ij} \sim \epsilon ^e_i \epsilon ^e_j \sim \epsilon ^{E_i + E_j }. \end{aligned}$$

66 $$\begin{aligned} (\delta _{LR}^e)_{ij}&\sim \frac{v_d A g_\rho }{\tilde{m}_L \tilde{m}_E} \epsilon ^\ell _i \epsilon ^e_j \sim \frac{m^e_i A}{\tilde{m}_L \tilde{m}_E}\epsilon ^{E_j - E_i} \sim \frac{m^e_j A}{\tilde{m}_L \tilde{m}_E}\epsilon ^{L_i - L_j}.\nonumber \\ \end{aligned}$$

In PC, the leading contributions to BR$(\mu \rightarrow e\gamma )$ typically arise from $(\delta _{LR}^e)_{12}$. In particular, in the anarchical scenario we find

67 $$\begin{aligned} \mathrm{BR}(\mu \rightarrow e \gamma )&\sim 6 \times 10^{-13}\left( \frac{5~\mathrm{TeV}}{{\tilde{m}}}\right) ^4, \end{aligned}$$

while in the hierarchical case we have a mild additional suppression by a factor of $\epsilon ^2_H \approx 0.1$.

Note, however, that in PC the left-handed “charges” $L_i$ are determined from the PMNS matrix analogously to $U(1)$ models only in the case of light Dirac neutrinos. If instead light neutrinos are Majorana, then the Weinberg operator can arise from a bilinear coupling to the composite sector (instead of linear couplings that resemble the $U(1)$ structure). In this case only the combination $L_i + E_j$ is determined by charged-lepton Yukawa couplings, and the constraints from LFV can be significantly relaxed by choosing symmetric charges [34]

68 $$\begin{aligned} \epsilon ^{L_i} \sim \epsilon ^{E_i} \sim \sqrt{\frac{y_i^e}{g_\rho }}. \end{aligned}$$

This implies

69 $$\begin{aligned} \frac{\tilde{m}}{m_\mu } (\delta _{LR}^e)_{12} \sim \epsilon ^{L_1 - L_2} \sim \sqrt{\frac{m_e}{m_\mu }}, \end{aligned}$$

and thus

70 $$\begin{aligned} \mathrm{BR}(\mu \rightarrow e \gamma ) \sim 7 \times 10^{-13}\left( \frac{1.5~\mathrm{TeV}}{{\tilde{m}}}\right) ^4. \end{aligned}$$

On the other hand, the predictions for the electron EDM are completely independent of any charge assignments since in PC the diagonal elements of the A-terms are generally complex. As a result, we find

71 $$\begin{aligned} |d_e|&\sim 7 \times 10^{-29} \left( \frac{3~\mathrm{TeV}}{{\tilde{m}}}\right) ^2 \mathrm{Im} \left( \frac{M_1 A}{\tilde{m}^2}\right) ~\mathrm{e~cm}, \end{aligned}$$

and therefore the eEDM now provides the strongest constraint on the PC scenario. Notice that the electron EDM has a similar sensitivity to NP effects in PC scenarios and $U(1)$ flavor models, independently of the particular charge assignments, pushing the SUSY scale to ${\tilde{m}} \gtrsim 3 \div 5 ~\mathrm{TeV}$. Needless to say, neither PC scenarios nor SUGRA with an underlying $U(1)$ flavor model can explain the muon $g-2$ anomaly.

In Table 2, we summarize the predictions for the MIs most relevant for phenomenology in various models: SUGRA (first column), PC (second column) and FGM (last column). On general ground, comparing the flavor structure of the soft sector of SUGRA and PC/FGM scenarios, the most prominent feature is the higher suppression for off-diagonal sfermion masses in the LL and RR sectors in the PC/FGM case. The LR sector has the same parametric structure in PC and SUGRA, since in both scenarios the A-terms are proportional to the SM Yukawas, while in FGM we have a much stronger suppression arising from a partial alignment among SM Yukawas and A-terms. Finally, PC and SUGRA share also the same SUSY CP problem as they allow complex diagonal elements for the A-terms. In contrast, within FGM, the leading CPV phases arise only at higher order in MI expansions and therefore are very suppressed.

**Table 2:** Predictions for the mass insertions in various SUSY models with an underlying $U(1)$ flavor model where $L_i$ ($E_i$) stands for the charges of $SU(2)$ doublets (singlets). Note that for the sake of simplicity we compare only single mass insertions, for large $\tan \beta$ triple mass insertions can possibly give the dominant contributions to LR transitions

	SUGRA $U(1)$	PC	FGM $U(1)$
$\frac{\tilde{m}}{m_e} \mathrm{Im} (\delta _{LR})_{11}$	$1$	$1$	$y_\tau ^4 t_\beta$
$\frac{\tilde{m}}{m_\mu } (\delta _{LR})_{12}$	$\epsilon ^{L_{1}-L_{2}}$	$\epsilon ^{L_{1}-L_{2}}$	$y_\tau ^2 \epsilon ^{L_{1}+L_{2}-2 L_{3}}$
$\frac{\tilde{m}}{m_\mu } (\delta _{LR})_{21}$	$\epsilon ^{E_1 - E_2}$	$\epsilon ^{E_1 - E_2}$	$y_\tau ^2 \epsilon ^{ E_1 - E_2 + 2 L_1 - 2 L_3}$
$(\delta _{LL})_{12}$	$\epsilon ^{L_{1} - L_{2}}$	$\epsilon ^{L_{1} + L_{2}}$	$y_\tau ^2 \epsilon ^{L_{1} + L_{2} -2 L_{3}}$
$(\delta _{RR})_{12}$	$\epsilon ^{E_1 - E_2}$	$\epsilon ^{E_{1} + E_{2}}$	$y_\tau ^2 \epsilon ^{E_1 + E_2 - 2E_3}$

## Conclusions

Now that the Higgs boson has been discovered, naturalness becomes a pressing question waiting for the final answer of LHC14. If new dynamics is present around the TeV scale, as needed to explain the smallness of the electroweak scale, one would expect too large contributions to flavor transitions mediated by the new physics states unless some protection mechanism is at work. Therefore, the possibility of finding new physics at the LHC is closely related to the existence of a suppression of flavor violating processes.

In this respect, MGM provides an ideal framework to accomplish this job. Indeed, if the flavor scale is much higher than the SUSY messenger scale then the flavor structure of the soft terms is entirely determined by the SM Yukawas, thus realizing the paradigm of MFV [15]. The drawback of this scenario is that any imprint of the flavor sector in low-energy physics and thus the possibility to test the flavor dynamics is completely lost.

On the other hand, minimal realizations of GMSB are now seriously challenged by the Higgs boson discovery at the LHC, since they can account for $m_h\approx 126$ GeV only at the price of a SUSY spectrum that is beyond the reach of the LHC. This has motivated extensions of minimal GMSB models by introducing new direct couplings between the messengers and the MSSM matter fields in order to obtain a large Higgs mass for light stops by generating non-vanishing A-terms at the messenger scale [17–27].

Among these scenarios, FGM [16] assumes that these new couplings have a flavor structure which is controlled by the same underlying flavor symmetry that explains the smallness of Yukawa couplings. As a result, FGM allows one to generate soft masses which still carry information about the high-scale flavor sector. Interestingly, due to the loop origin of soft terms, sfermion masses exhibit a flavor pattern that is much stronger suppressed than in gravity mediation. This strong suppression, arising even in the context of single $U(1)$ flavor models, is reminiscent of what happens in the case of wave function renormalization [29, 30] or Partial Compositeness [31–34]. In addition there is a strong suppression of LR flavor transitions that is particularly effective for accompanying flavor-blind phases, thus rendering the strong bounds from EDMs under control. Therefore FGM does not only modify the SUSY spectrum of MGM in a way interesting for collider phenomenology, but it also allows one to obtain a rich flavor phenomenology beyond MFV. In particular it offers a viable SUSY implementation of simple U(1) flavor symmetry models that in the context of gravity mediation have huge difficulties in passing the bounds from precision observables, and in the context of MGM are not testable at all.

While in Ref. [28] we concentrated on the quark sector, in this work we have focused on the lepton sector analyzing the implications of FGM with underlying $U(1)$ flavor models. In particular, we have studied the predictions of two models (the *anarchical* and *hierarchical* scenarios of Ref. [10]) that are representative for a whole class of $U(1)$ models that accommodate lepton masses and mixing angles. We have analyzed $\mu \rightarrow e \gamma$ (which turned out to be the most constraining LFV channel), the electron EDM $d_e$ and the muon anomalous magnetic moment $\Delta a_\mu$. Since the experimental bounds on both $\mu \rightarrow e \gamma$ and $d_e$ underwent recently an impressive improvement, an important question of this work was to establish whether and to which extent single $U(1)$ flavor models were viable in the context of FGM models. A related relevant question was to establish whether the current muon $g-2$ anomaly could be resolved while being compatible with the LFV and EDM bounds.

In the following, we summarize our main findings:

- The non-holomorphic soft masses (LL and RR mass insertions) are suppressed by powers of the spurion that are the *sum* of U(1) charges, in contrast to the corresponding gravity-mediated case where charge differences enter. The origin of this suppression is due to the fact that the $U(1)$ controls soft terms only indirectly via the messenger sector, which in turn generates soft terms only at loop level, thus leading to a double suppression by small couplings. This strong suppression is similar to the cases of wave function renormalization or SUSY partial compositeness.
- The A-terms are much more suppressed than in PC and gravity-mediated scenarios where they are proportional to the leading-order term $\epsilon ^{L_i + E_j}$ allowed by the $U(1)$ symmetry. Moreover, the A-terms are partially aligned to the Yukawa couplings and their diagonal components are real therefore not inducing contributions for the EDM. The first non-vanishing CP violating phase in the diagonal A-terms can arise only through higher-order expansions in the MIs, which leads to an additional suppression by powers of $y_\tau$ that becomes particularly effective for low $\tan \beta$.
- LFV processes and the electron EDM can be kept under control even for a light spectrum well below the TeV scale, provided that $\tan \beta$ is small (the smaller the better). This is true both in the *anarchical* and especially in the *hierarchical* scenarios. In contrast, PC and gravity-mediated scenarios require a very heavy spectrum above the TeV scale in order to fulfill the experimental bounds on BR$(\mu \rightarrow e\gamma )$ and $d_e$. Very low values for $\tan \beta$ also perfectly fit a complete realization of this setup within the NMSSM, which is the natural choice to generate the $\mu$-term in FGM scenarios, solving also the $\mu -B_\mu$ problem of MGM [21, 38].
- In spite of the tremendous experimental bounds on the electron EDM and LFV processes, we have found that it is still possible to account for the muon $g-2$ anomaly within FGM models in the *hierarchical* but not in the *anarchical* scenarios. The same conclusion is not true in PC and gravity-mediated models, where both $\mu \rightarrow e\gamma$ and $d_e$ prevent any sizable effect in $\Delta a_\mu$.
- Although BR$(\mu \rightarrow e\gamma )$ and $d_e$ have comparable sensitivities to FGM scenarios, $\mu \rightarrow e\gamma$ is currently more constraining. However, considering the slower decoupling of NP effects in $d_e \sim {\tilde{m}}^{-2}$ with respect to BR$(\mu \rightarrow e\gamma )\sim {\tilde{m}}^{-4}$, the electron EDM might become the most powerful probe of the scenarios in question with improved experimental data especially in the case of heavy superpartners.

In conclusion, we have analyzed in detail the anatomy and phenomenology in the lepton sector of FGM models with underlying $U(1)$ models. Remarkably, it turned out that these models can pass the impressive bounds on LFV processes and leptonic EDMs even for light superpartners, potentially observable at LHC14, leaving open the possibility of accommodating the longstanding muon $(g-2)$ anomaly and testing $U(1)$ flavor models in upcoming experiments.

## Appendix A: Formulas for the leptonic dipoles

In this appendix we collect the formulae for LFV branching ratios BR$(l_i \rightarrow l_j \gamma )$, lepton anomalous magnetic moments $\Delta a_i$, and lepton electric dipole moments $d_i$. The results have been obtained from the exact results of Refs. [65, 66] using a generalized mass insertion approximation (see e.g. Ref. [67]) without assuming large $\tan \beta$.

### A.1 Lepton flavor violation: $l_i \rightarrow l_j \gamma$

The branching ratio $\mathrm{BR}(l_i \rightarrow l_j \gamma )$ is given by

72 $$\begin{aligned} \frac{\mathrm{BR}(l_i \rightarrow l_j \gamma )}{\mathrm{BR}(l_i \rightarrow l_j \nu _i \overline{\nu }_j)} = \frac{48 \pi ^3 \alpha }{G_F^2} \left( |A^{ij}_{L}|^2 + |A^{ij}_{R}|^2 \right) . \end{aligned}$$

The amplitude $A^{ij}_L$ receives a neutralino and chargino contribution $A^{ij}_L = A^{ij(n)}_L + A^{ij(c)}_L$, whereas $A^{ij}_R$ receives only a neutralino contribution $A^{ij}_R = A^{ij(n)}_R$. These contributions are given by

73 $$\begin{aligned} A^{ij(n)}_L&= \frac{\alpha _2}{4 \pi } \frac{(m_{LL}^2)_{ij}}{m_L^4}\nonumber \\&\quad \times \left[ f_{1n} (x_{2L}) + \frac{(|M_2|^2 + \mu M_2 t_\beta )}{|M_2|^2 - |\mu |^2} f_{2n} (x_{2L}) \right. \nonumber \\&\quad - \left. \frac{(|\mu |^2 + \mu M_2 t_\beta )}{|M_2|^2 - |\mu |^2} f_{2n} (x_{\mu L}) \right] + \frac{\alpha _Y}{4 \pi } \frac{(m_{LL}^2)_{ij}}{m_L^4}\nonumber \\&\quad \times \left[ f_{1n} (x_{1L}) - \frac{(|M_1|^2 + \mu M_1 t_\beta )}{|M_1|^2 - |\mu |^2} f_{2n} (x_{1L})\right. \nonumber \\&\quad \left. + \frac{(|\mu |^2 + \mu M_1 t_\beta )}{|M_1|^2 - |\mu |^2} f_{2n} (x_{\mu L}) \right] \nonumber \\&\quad - \frac{\alpha _Y}{4 \pi } \frac{M_1}{m_{\mu }} \frac{(m_{LR}^2)_{ji}^*}{m_L^2 - m_R^2}\nonumber \\&\quad \times \left[ \frac{1}{m_L^2} f_{3n} (x_{1L}) - \frac{1}{m_R^2} f_{3n} (x_{1R}) \right] \nonumber \\&\quad - \frac{\alpha _Y}{4 \pi } \frac{M_1}{m_{\mu }} \frac{(m_{LR}^2 m_{RR}^2)_{ji}^*}{(m_L^2 - m_R^2) m_R^2}\nonumber \\&\quad \times \left[ \frac{m_R^2}{m_L^2 - m_R^2} \left( \frac{1}{m_L^2} f_{3n} (x_{1L}) - \frac{1}{m_R^2} f_{3n} (x_{1R}) \right) \right. \nonumber \\&\quad \left. + \frac{2}{m_R^2} f_{2n}(x_{1R}) \right] \nonumber \\&\quad + \frac{\alpha _Y}{4 \pi } \frac{M_1}{m_{\mu }} \frac{(m_{LL}^2 m_{LR}^2)_{ji}^*}{(m_L^2 - m_R^2) m_L^2}\nonumber \\&\quad \times \left[ \frac{m_L^2}{m_L^2 - m_R^2} \left( \frac{1}{m_L^2} f_{3n} (x_{1L}) - \frac{1}{m_R^2} f_{3n} (x_{1R}) \right) \right. \nonumber \\&\quad \left. + \frac{2}{m_L^2} f_{2n}(x_{1L}) \right] \nonumber \\&\quad + \frac{\alpha _Y}{2 \pi } \frac{M_1}{m_{\mu }} \frac{(m_{LL}^2 m_{LR}^2 m_{RR}^2)_{ji}^*}{(m_L^2 - m_R^2)^2 m_L^2 m_R^2} \nonumber \\&\quad \times \left[ \frac{m_L^2 m_R^2}{m_L^2 - m_R^2} \left( \frac{1}{m_L^2} f_{3n} (x_{1L}) - \frac{1}{m_R^2} f_{3n} (x_{1R}) \right) D\right. \nonumber \\&\quad \left. + \frac{m_R^2}{m_L^2} f_{2n}(x_{1L}) + \frac{m_L^2}{m_R^2} f_{2n}(x_{1R}) \right] , \end{aligned}$$

74 $$\begin{aligned} A^{ij(c)}_L&= \frac{\alpha _2}{4 \pi } \frac{(m_{LL}^2)_{ij}}{m_L^4}\nonumber \\&\quad \times \left[ f_{1c} (x_{2L}) + \frac{(|M_2|^2 + \mu M_2 t_\beta )}{|M_2|^2 - |\mu |^2} f_{2c} (x_{2L})\right. \nonumber \\&\quad \left. - \frac{(|\mu |^2 + \mu M_2 \tan \beta )}{|M_2|^2 - |\mu |^2} f_{2c} (x_{\mu L}) \right] , \end{aligned}$$

75 $$\begin{aligned} A^{ij(n)}_R&= \frac{\alpha _Y}{2 \pi } \frac{(m_{RR}^2)_{ij}}{m_R^4}\nonumber \\&\quad \times \left[ 2 f_{1n} (x_{1R}) + \frac{(|M_1|^2 + \mu M_1 t_\beta )}{|M_1|^2 - |\mu |^2} f_{2n} (x_{1R}) \nonumber \right. \\&\quad -\left. \frac{(|\mu |^2 + \mu M_1 t_\beta )}{|M_1|^2 - |\mu |^2} f_{2n} (x_{\mu R}) \right] - \frac{\alpha _Y}{4 \pi } \frac{M_1}{m_{\mu }} \frac{(m_{LR}^2)_{ij}}{m_L^2 - m_R^2}\nonumber \\&\quad \times \left[ \frac{1}{m_L^2} f_{3n} (x_{1L}) - \frac{1}{m_R^2} f_{3n} (x_{1R}) \right] \nonumber \\&\quad - \frac{\alpha _Y}{4 \pi } \frac{M_1}{m_{\mu }} \frac{(m_{LR}^2 m_{RR}^2)_{ij}}{(m_L^2 - m_R^2) m_R^2}\nonumber \\&\quad \times \left[ \frac{m_R^2}{m_L^2 - m_R^2} \left( \frac{1}{m_L^2} f_{3n} (x_{1L}) - \frac{1}{m_R^2} f_{3n} (x_{1R}) \right) \right. \nonumber \\&\quad \left. + \frac{2}{m_R^2} f_{2n}(x_{1R}) \right] + \frac{\alpha _Y}{4 \pi } \frac{M_1}{m_{\mu }} \frac{(m_{LL}^2 m_{LR}^2)_{ij}}{(m_L^2 - m_R^2) m_L^2}\nonumber \\&\quad \times \left[ \frac{m_L^2}{m_L^2 - m_R^2} \left( \frac{1}{m_L^2} f_{3n} (x_{1L})\right. \right. \nonumber \\&\quad \left. \left. - \frac{1}{m_R^2} f_{3n} (x_{1R}) \right) + \frac{2}{m_L^2} f_{2n}(x_{1L}) \right] \nonumber \\&\quad + \frac{\alpha _Y}{2 \pi } \frac{M_1}{m_{\mu }} \frac{(m_{LL}^2 m_{LR}^2 m_{RR}^2)_{ij}}{(m_L^2 - m_R^2)^2 m_L^2 m_R^2} \nonumber \\&\quad \times \left[ \frac{m_L^2 m_R^2}{m_L^2 - m_R^2} \left( \frac{1}{m_L^2} f_{3n} (x_{1L}) - \frac{1}{m_R^2} f_{3n} (x_{1R}) \right) \right. \nonumber \\&\quad \left. + \frac{m_R^2}{m_L^2} f_{2n}(x_{1L}) + \frac{m_L^2}{m_R^2} f_{2n}(x_{1R}) \right] . \end{aligned}$$

Here $(m_{LR}^2)_{ii} = m_{l_i}(A_i - \mu ^* t_\beta )$, $x_{iA} = |M^2_i|/m^2_A$, $x_{\mu A} = |\mu |^2/m^2_A$ with $i=1,2$ and $A=L,R$.

The explicit expressions for the loop functions are

76 $$\begin{aligned} f_{1n}(x)&= \frac{-17x^3+9x^2+9x-1+6x^2(x+3)\ln x}{24(1-x)^5},\end{aligned}$$

77 $$\begin{aligned} f_{2n}(x)&= \frac{-5x^2+4x+1+2x(x+2)\ln x}{4(1-x)^4},\end{aligned}$$

78 $$\begin{aligned} f_{3n}(x)&= \frac{1+2x\ln x-x^2}{2(1-x)^3},\end{aligned}$$

79 $$\begin{aligned} f_{1c}(x)&= \frac{-x^3-9x^2+9x+1+6x(x+1)\ln x}{6(1-x)^5},\end{aligned}$$

80 $$\begin{aligned} \ f_{2c}(x)&= \frac{-x^2-4x+5+2(2x+1)\ln x}{2(1-x)^4}. \end{aligned}$$

In the degenerate SUSY limit $M_1 = M_2 = \mu = m_L = m_R = \tilde{m}$ one obtains

81 $$\begin{aligned} A^{ij(n)}_L&= - \frac{\alpha _2}{120 \pi } \frac{(m_{LL}^2)_{ij}}{\tilde{m}^4} \left[ \frac{1}{8} + t_\beta \right] + \frac{\alpha _Y}{120 \pi } \frac{(m_{LL}^2)_{ij}}{\tilde{m}^4}\nonumber \\&\quad \times \left[ - \frac{5}{8} + t_\beta \right] + \frac{\alpha _Y}{48 \pi } \frac{\tilde{m}}{m_{l_i}} \frac{(m_{LR}^2)_{ji}^*}{\tilde{m}^4} \nonumber \\&\quad - \frac{\alpha _Y}{80 \pi } \frac{\tilde{m}}{m_{l_i}} \left[ \frac{(m_{LR}^2 m_{RR}^2)_{ji}^*}{\tilde{m}^6} + \frac{(m_{LL}^2 m_{LR}^2)_{ji}^*}{\tilde{m}^6} \right. \nonumber \\&\quad -\left. \frac{2}{3} \frac{(m_{LL}^2 m_{LR}^2 m_{RR}^2)_{ji}^*}{\tilde{m}^8} \right] ,\end{aligned}$$

82 $$\begin{aligned} A^{ij(c)}_L&= \frac{\alpha _2}{40 \pi } \frac{(m_{LL}^2)_{ij}}{\tilde{m}^4} \left[ \frac{1}{3} + t_\beta \right] , \end{aligned}$$

83 $$\begin{aligned} A^{(n)}_R&= - \frac{\alpha _Y}{60 \pi } \frac{(m_{RR}^2)_{ij}}{\tilde{m}^4} \left[ \frac{1}{2} + t_\beta \right] \nonumber \\&\quad + \frac{\alpha _Y}{48 \pi } \frac{\tilde{m}}{m_{l_i}} \frac{(m_{LR}^2)_{ij}}{\tilde{m}^4} - \frac{\alpha _Y}{80 \pi } \frac{\tilde{m}}{m_{l_i}} \frac{(m_{LR}^2 m_{RR}^2)_{ij}}{\tilde{m}^6 } \nonumber \\&\quad - \frac{\alpha _Y}{80 \pi } \frac{\tilde{m}}{m_{l_i}} \frac{(m_{LL}^2 m_{LR}^2)_{ij}}{\tilde{m}^6} + \frac{\alpha _Y}{120 \pi } \frac{\tilde{m}}{m_{l_i}} \frac{(m_{LL}^2 m_{LR}^2 m_{RR}^2)_{ij}}{ \tilde{m}^8}. \end{aligned}$$

### A.2 Anomalous magnetic moments

The supersymmetric contributions to the anomalous magnetic moment $\Delta a_{l_i}$ come from neutralino and chargino loops $\Delta a_{l_i} = \Delta a^{(n)}_{l_i} + \Delta a^{(c)}_{l_i}$. They read

84 $$\begin{aligned} \Delta a^{(n)}_{l_i}&= \frac{\alpha _2 }{ 8 \pi } \frac{m_{l_i}^2}{m_L^2}\nonumber \\&\quad \times \left[ - f_n^L (x_{2L})+ 2 \frac{|M_2|^2 + \mathrm{Re}(\mu M_2) \, t_\beta }{|M_2|^2 - |\mu |^2} f_{3n} (x_{2L})\right. \nonumber \\&\quad \left. - 2 \frac{|\mu |^2 + \mathrm{Re}(\mu M_2) \, t_\beta }{|M_2|^2 - |\mu |^2} f_{3n} (x_{\mu L}) \right] - \frac{\alpha _Y}{ 2 \pi } \frac{m_{l_i}^2}{m_R^2}\nonumber \\&\quad \times \left[ f_n^L(x_{1R}) - \frac{|M_1|^2 + \mathrm{Re}(\mu M_1) \, t_\beta }{|M_1|^2 - |\mu |^2} f_{3n} (x_{1R})\right. \nonumber \\&\quad \left. + \frac{|\mu |^2 + \mathrm{Re} (\mu M_1) \, t_\beta }{|M_1|^2 - |\mu |^2} f_{3n} (x_{\mu R}) \right] + \frac{\alpha _Y}{ 8 \pi } \frac{m_{l_i}^2}{m_L^2}\nonumber \\&\quad \times \left[ - f_n^L (x_{1L})- 2 \frac{|M_1|^2 + \mathrm{Re}(\mu M_1) \, t_\beta }{|M_1|^2 - |\mu |^2} f_{3n} (x_{1L})\right. \nonumber \\&\quad \left. + 2 \frac{|\mu |^2 + \mathrm{Re}(\mu M_1) \, t_\beta }{|M_1|^2 - |\mu |^2} f_{3n} (x_{\mu L}) \right] \nonumber \\&\quad + \frac{\alpha _Y}{ 2 \pi } \frac{m_{l_i}}{m_L^2 - m_R^2} \mathrm{Re}(M_1 m_{LR}^2)_{ii}\nonumber \\&\quad \times \left[ \frac{1}{m_L^2} f_{3n}(x_{1L}) - \frac{1}{m_R^2} f_{3n}(x_{1R}) \right] \nonumber \\&\quad + \frac{\alpha _Y}{ 2 \pi } \frac{m_{l_i}}{m_L^2 - m_R^2} \mathrm{Re}(M_1 m^2_{LR} m_{RR}^2)_{ii}\nonumber \\&\quad \times \left[ \frac{\frac{1}{m_L^2} f_{3n}(x_{1L}) - \frac{1}{m_R^2} f_{3n}(x_{1R})}{m_L^2 - m_R^2} + 2 \frac{1}{m_R^4}f_{2n}(x_{1R}) \right] \nonumber \\&\quad - \frac{\alpha _Y}{ 2 \pi } \frac{m_{l_i}}{m_L^2 - m_R^2} \mathrm{Re}(M_1 m^2_{LL} m_{LR}^2)_{ii}\nonumber \\&\quad \times \left[ \frac{\frac{1}{m_L^2} f_{3n}(x_{1L}) - \frac{1}{m_R^2} f_{3n} (x_{1R})}{m_L^2 - m_R^2} +2 \frac{1}{m_L^4} f_{2n}(x_{1L}) \right] \nonumber \\&\quad - \frac{\alpha _Y}{\pi }m_{l_i} \frac{\mathrm{Re}(M_1 m^2_{LL} m^2_{LR} m_{RR}^2)_{ii}}{(m_L^2 - m_R^2)^2}\nonumber \\&\quad \times \left[ \frac{\frac{1}{m_L^2}f_{3n}(x_{1L}) - \frac{1}{m_R^2}f_{3n}(x_{1R})}{m_L^2 - m_R^2}\!+\! \frac{f_{2n}(x_{1L})}{m_L^4} + \frac{f_{2n} (x_{1R})}{m_R^4} \right] ,\end{aligned}$$

85 $$\begin{aligned} \Delta a^{(c)}_{l_i}&=\frac{\alpha _2}{ 4 \pi } \frac{m_{l_i}^2}{m_L^2}\nonumber \\&\quad \times \left[ f^L_c (x_{2L}) - \frac{|M_2|^2 + \mathrm{Re}(\mu M_2) \, t_\beta }{|M_2|^2-|\mu |^2} f_c^{LR} (x_{2L})\right. \nonumber \\&\quad \left. + \frac{|\mu |^2 + \mathrm{Re}(\mu M_2) \, t_\beta }{|M_2|^2-|\mu |^2} f_c^{LR} (x_{\mu L}) \right] . \end{aligned}$$

Here we introduced the additional loop functions:

86 $$\begin{aligned} f^L_n (x)&= \frac{1-6x+3x^2+2x^3-6x^2 \log x}{6 (1-x)^4}, \end{aligned}$$

87 $$\begin{aligned} f^L_c (x)&= \frac{2+3x-6x^2+x^3 + 6 x \log x}{6 (1-x)^4}, \end{aligned}$$

88 $$\begin{aligned} f^{LR}_c (x)&= \frac{-3 + 4 x - x^2- 2 \log x}{ (1-x)^3}. \end{aligned}$$

In the degenerate SUSY limit $M_1 = M_2 = \mu = m_L = m_R = \tilde{m}$ one obtains

89 $$\begin{aligned} \Delta a^{(n)}_{l_i}&= - \frac{\alpha _2 }{ 48 \pi } \frac{m_{l_i}^2}{\tilde{m}^2} \left[ - \frac{1}{2} + \frac{\mathrm{Re} (\mu M_2)}{\tilde{m}^2} t_\beta \right] -\frac{\alpha _Y}{48 \pi } \frac{m_{l_i}^2}{\tilde{m}^2}\nonumber \\&\quad \times \left[ \left( \frac{3}{2} + \frac{\mathrm{Re} (\mu M_1)}{\tilde{m}^2} t_\beta \right) +\frac{2\mathrm{Re} (M_1 m_{LR}^2)_{ii}}{m_{l_i}\tilde{m}^2} \right] \nonumber \\&\quad + \frac{\alpha _Y}{ 40 \pi } \frac{m_{l_i}}{\tilde{m}^2}\nonumber \\&\quad \times \left[ \frac{\mathrm{Re} (M_1 m^2_{LR} m_{RR}^2)_{ii}}{\tilde{m}^4} + \frac{\mathrm{Re} (M_1 m^2_{LL} m_{LR}^2)_{ii}}{\tilde{m}^4}\right. \nonumber \\&\quad \left. - \frac{2}{3} \frac{\mathrm{Re} (M_1 m^2_{LL} m^2_{LR} m_{RR}^2)_{ii}}{\tilde{m}^6} \right] ,\end{aligned}$$

90 $$\begin{aligned} \Delta a^{(c)}_{l_i}&= \frac{\alpha _2}{ 8 \pi } \frac{m_{l_i}^2}{\tilde{m}^2} \left[ - \frac{1}{6} + \frac{\mathrm{Re} (\mu M_2)}{\tilde{m}^2} t_\beta \right] . \end{aligned}$$

### A.3 Electric dipole moments

The supersymmetric contributions to the electric dipole moment $d_i$ come from neutralino and chargino loops $d_i = d^{(n)}_i + d^{(c)}_i$. They read

91 $$\begin{aligned} \frac{d^{(n)}_i}{e}&= \frac{\alpha _2 }{ 8 \pi } \frac{m_{l_i}}{m_L^2} \frac{ \mathrm{Im}(\mu M_2)}{|M_2|^2 - |\mu |^2} t_\beta \left[ f_{3n} (x_{2L}) - f_{3n}(x_{\mu L}) \right] \nonumber \\&\quad +\frac{\alpha _Y}{ 4 \pi } \frac{m_{l_i}}{m_R^2} \frac{\mathrm{Im}(\mu M_1)}{|M_1|^2 - |\mu |^2} t_\beta \left[ f_{3n}(x_{1R}) - f_{3n} (x_{\mu R}) \right] \nonumber \\&\quad - \frac{\alpha _Y}{ 8 \pi } \frac{m_{l_i}}{m_L^2} \frac{\mathrm{Im} (\mu M_1)}{|M_1|^2 - |\mu |^2} t_\beta \left[ f_{3n}(x_{1L}) - f_{3n}(x_{\mu L}) \right] \nonumber \\&\quad + \frac{\alpha _Y}{ 4 \pi } \frac{\mathrm{Im} (M_1 m_{LR}^2)_{ii}}{m_L^2 - m_R^2} \left[ \frac{1}{m_L^2} f_{3n}(x_{1L}) - \frac{1}{m_R^2} f_{3n}(x_{1R}) \right] \nonumber \\&\quad + \frac{\alpha _Y}{ 4 \pi } \frac{\mathrm{Im}(M_1 m^2_{LR} m_{RR}^2)_{ii}}{m_L^2 - m_R^2} \nonumber \\&\quad \times \left[ \frac{\frac{1}{m_L^2} f_{3n}(x_{1L}) - \frac{1}{m_R^2} f_{3n}(x_{1R})}{m_L^2 - m_R^2} + 2 \frac{1}{m_R^4} f_{2n} (x_{1R}) \right] \nonumber \\&\quad - \frac{\alpha _Y}{ 4 \pi } \frac{\mathrm{Im} (M_1 m^2_{LL} m_{LR}^2)_{ii}}{m_L^2 - m_R^2} \nonumber \\&\quad \times \left[ \frac{\frac{1}{m_L^2} f_{3n}(x_{1L}) - \frac{1}{m_R^2} f_{3n}(x_{1R})}{m_L^2 - m_R^2} + 2 \frac{1}{m_L^4} f_{2n} (x_{1L}) \right] \nonumber \\&\quad - \frac{\alpha _Y}{ 2 \pi } \frac{ \mathrm{Im} (M_1 m^2_{LL} m^2_{LR} m_{RR}^2)_{ii}}{(m_L^2 - m_R^2)^2} \nonumber \\&\quad \times \left[ \frac{\frac{1}{m_L^2}f_{3n}(x_{1L}) - \frac{1}{m_R^2} f_{3n}(x_{1R})}{m_L^2 - m_R^2} + \frac{1}{m_L^4} f_{2n}(x_{1L}) \right. \nonumber \\&\quad \left. + \frac{1}{m_R^4} f_{2n}(x_{1R}) \right] , \end{aligned}$$

92 $$\begin{aligned} \frac{d^{(c)}_i}{e}&= - \frac{\alpha _2}{8\pi } \frac{m_{l_i}}{m_L^2} \frac{\mathrm{Im} (\mu M_2)}{|M_2|^2-|\mu |^2} t_\beta \left[ f_c^{LR} (x_{2L}) - f_c^{LR} (x_{\mu L}) \right] . \end{aligned}$$

In the degenerate SUSY limit $M_1 = M_2 = \mu = m_L = m_R = \tilde{m}$ one obtains

93 $$\begin{aligned} \frac{d^{(n)}_i}{e}&= - \frac{\alpha _2 }{ 96 \pi } \frac{m_{l_i}}{\tilde{m}^2} \frac{ \mathrm{Im} (\mu M_2)}{\tilde{m}^2} t_\beta - \frac{\alpha _Y}{ 96 \pi } \frac{m_{l_i}}{\tilde{m}^2} \frac{ \mathrm{Im} (\mu M_1)}{\tilde{m}^2} t_\beta \nonumber \\&\quad - \frac{\alpha _Y}{ 120 \pi } \frac{\mathrm{Im} (M_1 m^2_{LL} m^2_{LR} m_{RR}^2)_{ii}}{\tilde{m}^8}\nonumber \\&\quad - \frac{\alpha _Y}{ 48 \pi } \frac{\mathrm{Im} (M_1 m_{LR}^2)_{ii}}{\tilde{m}^4} + \frac{\alpha _Y}{ 80 \pi } \frac{\mathrm{Im} (M_1 m^2_{LR} m_{RR}^2)_{ii}}{\tilde{m}^6} \nonumber \\&\quad + \frac{\alpha _Y}{ 80 \pi } \frac{\mathrm{Im} (M_1 m^2_{LL} m_{LR}^2)_{ii}}{\tilde{m}^6},\end{aligned}$$

94 $$\begin{aligned} \frac{d^{(c)}_i}{e}&= \frac{\alpha _2}{ 16 \pi } \frac{m_{l_i}}{\tilde{m}^2} \frac{ \mathrm{Im} (\mu M_2)}{\tilde{m}^2} t_\beta . \end{aligned}$$

## Footnotes

1 This conclusion holds even under the assumption of a mechanism inducing degenerate sfermion masses at the SUSY breaking mediation scale. In fact, the assumed flavor universality is broken by the renormalization group evolution of the soft masses down to the flavor breaking scale, so that large flavor mixing is anyway generated at the level predicted by the $U(1)$ symmetry [13].

2 Such matter-messenger couplings have recently received new interest, as they allow one to obtain a large Higgs mass with light stops by generating non-vanishing A-terms at the messenger scale [17–27].

3 For R-parity odd messengers similar couplings to Higgs fields are allowed and have been discussed in the literature, see e.g. [25]. Note that in this way one preserves the MFV structure of MGM. Here we are instead interested in non-trivial flavor structures.

4 We only consider terms in leading order in $\Lambda ^2/M^2$, i.e. a messenger scale that is not particularly low.

5 Notice that the $\mu$- and $B_\mu$-terms, which are not controlled by GMSB, could still introduce CP violating phases depending on the underlying mechanism that generates them. However, if this mechanism is such that the phases of $\mu$ and $B_\mu$ are correlated to the phase of $\Lambda$, then no phases arise from this sector [14].

6 We will eventually prefer low values for $\tan \beta$ ($\lesssim 5$). For the numerical analysis later on one has therefore to take into account all contributions, which are collected in the appendix. At this point we are rather interested in keeping the formulas simple and just give order-of-magnitude estimates.
